# Microbiome in radiotherapy: an emerging approach to enhance treatment efficacy and reduce tissue injury

**DOI:** 10.1186/s10020-024-00873-0

**Published:** 2024-07-19

**Authors:** Lina Lu, Fengxiao Li, Yuanyuan Gao, Shuhe Kang, Jia Li, Jinwang Guo

**Affiliations:** 1 School of Chemical Engineering, Northwest Minzu University, No.1, Northwest New Village, Lanzhou, Gansu 730030 China; 2 Key Laboratory of Environment-Friendly Composite Materials of the State Ethnic Affairs Commission, Lanzhou, Gansu China; 3 Gansu Provincial Biomass Function Composites Engineering Research Center, Lanzhou, Gansu China; 4 Key Laboratory for Utility of Environment-Friendly Composite Materials and Biomass in, University of Gansu Province, Lanzhou, Gansu China; 5 Department of Pharmacy, the Affiliated Hospital of Qingdao University, Qingdao, China; 6 Zibo Central Hospital, Zibo, Shandong China

**Keywords:** Radiotherapy, Injury, Microbiome, Efficacy, Probiotics, Metabolites, FMT

## Abstract

Radiotherapy is a widely used cancer treatment that utilizes powerful radiation to destroy cancer cells and shrink tumors. While radiation can be beneficial, it can also harm the healthy tissues surrounding the tumor. Recent research indicates that the microbiota, the collection of microorganisms in our body, may play a role in influencing the effectiveness and side effects of radiation therapy. Studies have shown that specific species of bacteria living in the stomach can influence the immune system’s response to radiation, potentially increasing the effectiveness of treatment. Additionally, the microbiota may contribute to adverse effects like radiation-induced diarrhea. A potential strategy to enhance radiotherapy outcomes and capitalize on the microbiome involves using probiotics. Probiotics are living microorganisms that offer health benefits when consumed in sufficient quantities. Several studies have indicated that probiotics have the potential to alter the composition of the gut microbiota, resulting in an enhanced immune response to radiation therapy and consequently improving the efficacy of the treatment. It is important to note that radiation can disrupt the natural balance of gut bacteria, resulting in increased intestinal permeability and inflammatory conditions. These disruptions can lead to adverse effects such as diarrhea and damage to the intestinal lining. The emerging field of radiotherapy microbiome research offers a promising avenue for optimizing cancer treatment outcomes. This paper aims to provide an overview of the human microbiome and its role in augmenting radiation effectiveness while minimizing damage.

**Supplementary Information:**

The online version contains supplementary material available at 10.1186/s10020-024-00873-0.

## Introduction

The term “microbiota” refers to the community of microorganisms that reside within and on the human body, while “microbiome” pertains to the genetic material of the microbiota at specific body locations [1]. Various anatomical sites, such as the skin, mucosa, digestive system, pulmonary system, urogenital tract, and mammary gland, are inhabited by microorganisms [2]. Several factors, including age, diet, lifestyle, hormonal changes, genetic predisposition, and underlying health conditions, influence the composition of an individual’s microbiome at any given time [3]. However, alterations in the human microbiota, known as dysbiosis, can potentially lead to life-threatening conditions [2]. The gut microbiota plays a crucial role in regulating epithelial growth, modifying metabolic traits, and activating innate immunity, among other fundamental biological functions [4]. Moreover, the microbiota defends the body against foreign pathogens through competitive colonization and the production of antimicrobial compounds, such as bacteriocins [5]. Surprisingly, bacteria can influence the tumor microenvironment, impacting the efficacy of cancer therapies [6, 7]. The gut microbiota has been found to affect the effectiveness of various treatment modalities, including surgery, chemotherapy, immunotherapy, and androgen deprivation therapy [8–10]. The involvement of the gut microbiota in radiosensitivity is a relatively new concept that has garnered significant interest [11].

Radiotherapy remains a widely employed cancer treatment in clinical practice [12, 13]. However, radiation can cause considerable damage to normal rapidly dividing cells, particularly the gastrointestinal (GI) epithelium, resulting in acute enteropathy characterized by symptoms such as nausea, vomiting, abdominal discomfort, and diarrhea [14]. Consequently, numerous strategies have been developed to mitigate the significant challenges and health issues associated with radiation therapy for cancer survivors [14]. The microbiota has been extensively studied for its role in modulating immune responses to cancer and maintaining gut homeostasis, leading to investigations into its potential to modify the outcomes of radiotherapy [15, 16]. Adverse radiation effects include dysbiosis and mucosal homeostasis disruption [17]. Ferreira and colleagues [18] examined the relationship between gut microbiota and radiation-induced enteropathy following pelvic irradiation. They found a strong correlation between bacterial diversity and acute radiation-induced enteropathy, as opposed to late enteropathy. The initial stages of enteropathy showed increased levels of microbiota-derived short-chain fatty acids (SCFAs), followed by a rise in the abundance of SCFA producers such as *Clostridium IV*, *Roseburia*, and *Phascolarctobacterium*, alongside decreased levels of cytokines that regulate microbiota homeostasis (e.g., interleukin (IL)-12, IL-15, and IL-16) [18]. Direct evidence of the role of microbiota in radiation syndromes has been demonstrated by the effectiveness of fecal microbiota transplantation (FMT) in protecting patients from radiation-induced injury. Ding et al. [19] conducted a recent pilot study that confirmed the safety and effectiveness of FMT in treating chronic radiation enteritis (CRE). Out of five patients with chronic CRE, three showed significant improvements, including relief from diarrhea, rectal bleeding, abdominal/rectal discomfort, and fecal incontinence, following FMT. Consequently, efforts have focused on modifying or preserving microorganisms and their integrity during radiation therapy, including the use of probiotics, pharmacological interventions, and FMT, among others [20, 21]. However, despite extensive research on the relationship between radiation and microbial alterations, much remains to be understood about the mechanisms and processes by which the microbiota governs the host’s response to radiation. As a result, this review focuses on the mechanism of microbiota and radiation side effects, the development of research on intestinal microbiota and its metabolites, and treatment via microbiota regulation.

## Overview of radiotherapy and radiation injury mechanism

Radiation therapy, sometimes referred to simply as “radiotherapy,” is a treatment method that uses beams of high-energy radiation to destroy tumor cells [22]. Radiation therapy is available in several forms, the most common of which is X-rays, although proton beam therapy is another option [22]. The main radiotherapy method involves killing cancer cells and reducing their capacity to multiply [22]. This might be accomplished directly by destroying the DNA or other critical cellular components or indirectly by producing free radicals that cause cellular harm. Unfortunately, normal cells, particularly those that proliferate on a regular basis, might be injured during radiation therapy (Table 1). This effect on normal cells may be reduced by accurately directing the radiation beam on the tumor and fractionating the total radiation dose to enable normal tissue renewal and repair [23].

**Table 1:** Overview of radiotherapy-induced injuries in various cancers

Type of cancer	Study	Radiation type	Injury	Description	Ref
-	Clinical and in vivo	40 Gy	Skin injury	The microbiota-metabolite axis has offered intriguing therapeutic options for the treatment of unfavorable adverse reactions and performs a crucial role in radiation-induced skin injury (RISI).	[265]
Oral cancer	Clinical and in vitro	52 and 72 Gy	Oral mucositis	Oral mucositis is caused by the therapy’s inhibition of the immune system and bacterial dysbiosis.	[266]
Head and neck cancer	Clinical trial	62.33 Gy	Mucositis	Improvements in patient satisfaction with oral health care programs are essential, as shown by the relationship between xerostomia and hygiene conditions and changes in oral biofilms in patients who had received radiation therapy.	[114]
Pelvic malignancies	In vivo	6 Gy	Behavioral and neuronal damage	substantial microbial dysbiosis and behavioral abnormalities were seen after pelvic irradiation, including discrete changes in the microbial diversity and a substantial drop in the locomotor impact and level of anxiety at each time point.	[149]
Pelvic cancer	Clinical trial	44–50 Gy	Fatigue and diarrhea	Diarrhea, a systemic inflammatory reaction, and pelvic radiotherapy-related tiredness in cancer patients were all caused by radiation-induced dysbiosis, which also led to pelvic radiation ailments.	[253]
-	In vivo	-	Dysbiosis	The findings showed that rectal radiation causes dysbiosis, which conveys radiation and inflammatory sensitivity, and they provide proof that interleukin-1β (IL-1β) at least partially mediates the microbial-induced radiation tissue injury.	[267]
Breast, Head and neck, Brain tumor, Esophageal, Cervix or endometrial, NSCLC, Colorectal, Prostate, and SCLC	Clinical trial	**-**	Bacterial, viral, and fungal infections	Patients who had radiation treatment together with chemotherapy were more likely to develop infections, which were linked to lymphopenia after the end of radiation therapy (EoRT) in these patients.	[268]
Upper-Torso Cancer	Clinical trial		*Influenza* viruses A, *Influenza* viruses B, *Streptococcus pneumonia*, *Haemophilus influenzae*, *Neisseria meningitidis* and *Staphylococcus aureus*	*The most frequent pathogens were N. meningitidis*,* H. influenzae*,* influenza viruses A and B*,* S. pneumoniae*,* and S. aureu*s.	[269]
Head and neck cancer	Clinical trial	**-**	*Candida* infection	According to the current research, *Candida albicans*, *Candida tropicalis*, and *Candida parapsilosis* make up 89% of all isolates.	[270]

Once ionizing radiation strips electrons from atoms and molecules, highly reactive ions and ion pairs known as reactive oxygen species (ROS) are created [24]. ROS are thought to be the primary cause of radiation-induced DNA damage in cells [25]. Radiation ionizes the plentiful water in the body, releasing hydroxyl ROS, which includes extremely reactive hydroxyl radicals [3]. Hydroxyl radicals are highly reactive chemicals that react violently with DNA, proteins, and lipids [24]. Repairing hydroxyl radical-induced mutations is critical before cell division or transcription to avoid the spread of damaging genetic material [24]. These radicals may also take hydrogen atoms from the methyl groups of thymine nucleic acids and participate in further double-bond events [26]. This type of damage is often seen in the primary structure of DNA, which consists of a straight line of nucleotides. Ionizing radiation has a particularly destructive impact on DNA and regulatory proteins [24]. Regardless of dose, ionizing radiation may cause DNA strand breaks (both single-stranded breaks (SSBs) and double-stranded breaks (DSBs) by disrupting chemical connections along the helical backbone. DSBs cause more genomic instability than SSBs and may result in cell death or defective DNA repair methods, such as mutagenic single-strand annealing [24]. According to prevalent beliefs, parenchymal and/or vascular endothelial cells are thought to be eliminated due to radiation damage to normal tissues. Several studies have been conducted to investigate if these cell types or their progenitors are the principal targets of radiation-induced tissue damage [27]. Boron neutron capture irradiation caused extensive vascular damage, demyelination, and white matter necrosis with restricted dosage to parenchymal glial cells to be a result of selectively irradiating the microvasculature employing a boron compound that was given intraperitoneally and did not cross the blood-brain barrier (BBB). This indicates an essential role of endothelial cell loss in developing this condition [27]. Endothelial cell damage does not play a role in the development of intestinal illness, as shown by the fact that targeted irradiation of the vascular endothelium does not affect the survival of mouse intestinal crypt stem cells [28, 29].

Recent molecular and cellular studies show that when vascular endothelial cells or tissue stem/progenitor cells die, additional reactive pathways are set off, leading to even more significant cell loss, tissue damage, fibrosis, necrosis, and impairments in function [27]. Following radiation administration to the tissues and organs, an immediate cascade of chemokines and cytokines is triggered, with the mediators produced in the affected tissues sustaining and amplifying the inflammatory response over extended periods, potentially leading to persistent inflammation and tissue damage. Among the many pro-inflammatory chemokines and cytokines that are excessively generated immediately after radiation exposure, IL-1, IL-6, tumor necrosis factor (TNF)-α, and transforming growth factor (TGF)-β play significant roles in the skin, lung, and brain responses. Additionally, C-X-C chemokine ligand 12 (CXCL-12), also known as stromal cell-derived factor-1 (SDF-1), and C-X-C chemokine receptor type 4 (CXCR4) are chemokines responsible for facilitating the entry of bone marrow-derived cells (BMDC) into irradiated tissue [27].

## Microbiome and radiotherapy efficacy

**Fig. 1:**
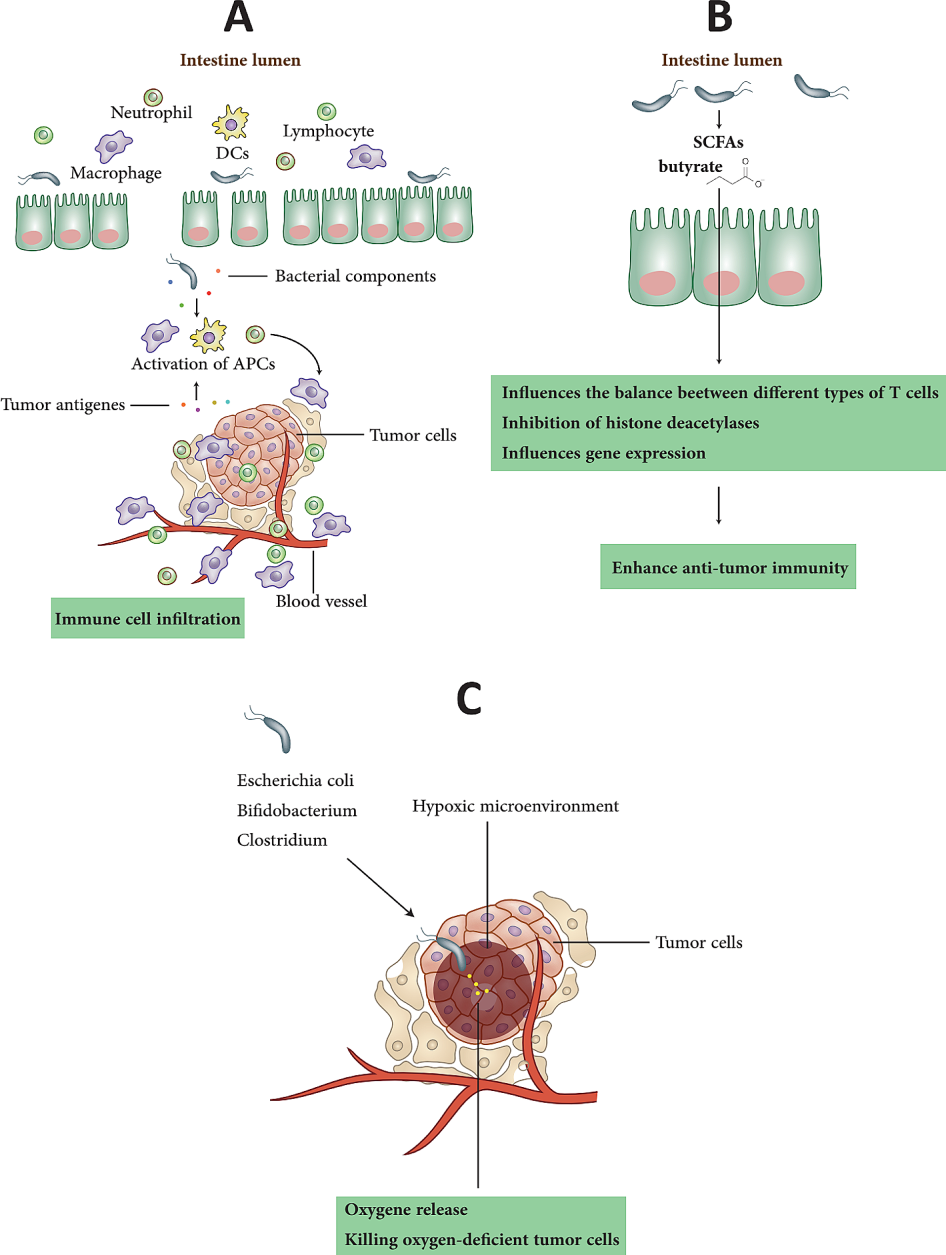
Microbiome and Radiotherapy Efficacy. The figure illustrates the impact of the microbiome on radiotherapy efficacy, highlighting the modulation of the immune response, the role of microbial metabolites, and strategies to improve tumor oxygenation and radio-sensitivity using bacteria. The figure is divided into three sections, each highlighting different aspects of the relationship between the microbiome and radiotherapy efficacy. (**A**) Microbiota modulates the immune response in radiotherapy: This section focuses on the ability of the microbiome to increase lymphocyte infiltration into tumors, leading to immunogenic cell death during radiotherapy. Additionally, it highlights the combined effect of bacterial components with tumor antigens in activating antigen-presenting cells and promoting immune cell infiltration. Bacterial-based immunotherapy, such as the use of bacterial outer membrane vesicles, is emphasized as a widely studied approach in combination with radiotherapy. Furthermore, the impact of the gut microflora, antibiotics like vancomycin, and dietary fiber on enhancing the antitumor immune response is discussed. The section also explores how microbial metabolites, specifically microbial butyrate and short-chain fatty acids (SCFAs), influence the expression of immune-related molecules and promote the differentiation of colonic Treg cells. (**B**) Microbial metabolites can enhance radiotherapy efficacy: It has been found the ability of microbial metabolites, particularly SCFAs like butyrate, to enhance the efficacy of radiotherapy by promoting anti-tumor immunity. The immunomodulatory properties of SCFAs, which affect the balance between tumor-killing CD4 + and CD8 + T cells and immune-suppressing Tregs, are found. Moreover, the inhibitory effect of butyrate on histone deacetylases (HDACs), crucial regulators of cell cycle regulation and proliferation, has been noted. The documents demonstrate the potent radiosensitizing effect of butyrate on colorectal cancer (CRC) through its impact on FOXO3A transcriptional activity and cell cycle arrest. (**C**) Microbiota and radio-sensitization by improving the tumor hypoxic microenvironment: This section explores innovative strategies to improve tumor oxygenation and radio-sensitivity by leveraging the microbiota. The combination therapy of *Bifidobacterium infantis* with its monoclonal antibody is presented as a successful approach to destroy the hypoxic tumor region, enhance radiation-induced DNA damage, and induce tumor cell apoptosis. The significance of relieving tumor hypoxia and improving the killing effect of radiotherapy is highlighted. Additionally, the section suggests an alternative approach to enhance tumor oxygenation by increasing blood perfusion at the tumor site, utilizing the local administration of Botulinum toxin-A. Furthermore, the role of bacteria and their components in improving tumor radio-sensitivity is discussed. This includes encoding enzymes that convert non-toxic prodrugs into radio-sensitizers and regulating the cell cycle to transition from a radio-resistant to a radio-sensitive phase

Several studies have suggested that the microbiota may affect radiation response (Table 2) [30]. For example, some studies have shown that certain types of bacteria, including *Firmicutes* and *Bacteroidetes* in the gut, can affect the immune system’s response to radiation, potentially enhancing the efficacy of the treatment [31]. Probiotics, which are living microorganisms that might give health advantages when ingested in sufficient proportions, are one possible pathway for leveraging the microbiome to increase radiotherapy effectiveness [32]. Some studies believe that probiotics may improve radiotherapy effectiveness by modifying the gut microbiota to induce an immunological response to radiation [33]. In this section, we overview and discuss the precise various roles and mechanisms of the microbiome on radiotherapy efficacy (Fig. 1).

**Table 2:** Effect of the microbiome on the promotion the radiotherapy efficacy

Microbiome based approach	Bacteria	Study setting	Type of cancer	Radiation type	Mechanism	Microbiota analyses	Outcome	Ref
Vancomycin	-	In vivo	Melanoma	21 Gy	Modulate dendritic cell (DC) antigen presentation, cytolytic CD8 + T cells	-	Through the cross-presentation of tumor-associated antigen to cytolytic CD8 + T cells and on Interferon-γ (IFN-γ), vancomycin enhanced the radiotherapy-induced anticancer immunity and tumor development suppression.	[271]
Butyrate	Lachnospiraceae	In vivo	Colorectal cancer (CRC)	-	Type I IFN expression	16 S rRNA sequencing	Local butyrate prevented TANK-binding kinase 1 (TBK1) and IFN regulatory factor 3 (IRF3) phosphorylation, preventing ionizing radiation-induced tumor-specific cytotoxic T-cell immunity without directly shielding tumor cells. This prevented the stimulator of IFN genes (STING) --activated type I IFN production in DCs, a type of immune cell.	[272]
Butyrate	-	Clinical trial	Prostate cancer	-	-	-	During T0 and T2, endoscopic assessment revealed that clinical state had improved in 50% of patients compared to 20% of controls; full normalization was seen in 10% of patients but not in any of the controls.	[273]
Oral microbiota transplantation	*Fusobacterium nucleatum*	In vivo	CRC	-	Gut bacterial composition	16 S rRNA sequencing	Metronidazole, an antibiotic that destroys Fusobacterium, has recently been identified as a possible radiosensitizer for treating cancers of the gastrointestinal (GI) tract.	[274]
Faecal microbiota transplantation (FMT)	-	In vitro and in vivo	Colorectal adenocarcinoma	1.0 Gy	Gut bacterial composition	16 S rRNA sequencing	The intestinal bacterial population and RNA expression profile of the mice exposed to radiation were both conserved by FMT.	[250]
Commensal bacteria	*Clostridiales*,* Lactobacillales* and *Burkholderiales*	In vivo	Breast cancer and melanoma	16 Gy	Antitumor T CD8 c + ell response and macrophage and Dectin-1	16 S rRNA sequencing	After tumors are exposed to radiation, commensal bacterial depletion causes commensal fungus to grow larger and reduces anticancer immunity. Through lowering macrophage-mediated immunosuppression, targeting commensal fungus improved the radiation-induced anticancer immune response.	[45]
Lycium barbarum	*Turicibacter* and *Akkermansia*	In vitro and in vivo	-	5.5, 6, and 8.5 Gy	Reconstituted the gut microbiota	16 S rRNA sequencing and LC-MS/MS	The improved production of immune-related cytokines and the quickening of lymphocyte recovery rates are the major characteristics of LBE’s positive immune-modulating effects.	[275]

### Microbiota modulates the immune response in response to radiotherapy

Several factors, including immunological modulation, play a crucial role in the growth and response of tumors to radiation [34]. Radiation therapy can enhance immunogenic cell death (ICD) and promote the infiltration of lymphocytes into the tumor [34]. Following radiotherapy, the tumor releases numerous tumor antigens, activating antigen-presenting cells and facilitating immune cell infiltration when combined with the immune-enhancing effects of certain bacterial or bacterial derivative compounds [35]. By combining radiotherapy with bacterial components that enhance immune system function in various ways, such as activating dendritic cell (DC) phenotypes, boosting the activity of cytotoxic T lymphocytes, and selectively eliminating myeloid-derived suppressor cells (MDSCs), an inflammatory environment is created, overcoming the limited systemic anti-tumor immune response typically observed with radiotherapy alone [36].

The most extensively studied approach combines radiation with bacterial-based immunotherapy [37]. Bacterial-related substances such as lipopolysaccharide (LPS), DNA, bacterial outer membrane vesicles (OMVs), peptidoglycan, and RNA stimulate DCs, macrophages, and neutrophils by engaging specific pattern recognition receptors (PRRs) via Toll-like receptors (TLRs) [38]. As a result, multiple targets within the cancer-immunity cycle are disrupted [36]. Of particular interest are the unmethylated CpG motifs present in bacterial DNA, which are responsible for inducing immunological activation [39]. Synthetic DNA sequences known as CpG oligodeoxynucleotides (ODNs) can be recognized by plasmacytoid DCs and B cells through the TLR9 receptor [40]. This signaling pathway through TLR9 is considered the least hazardous among TLR signaling pathways, leading to increased activation of DCs and higher expression of co-stimulatory molecules and major histocompatibility complexes (MHC) [41]. In an experiment involving mice, a single dose of 20 Gy radiation was administered either alone or in combination with active CpG ODN 1826 [42]. The results showed that the radiation environment resulted in more antigens from the dead tumor cells, which were taken up by the stimulated DCs, leading to specific T-cell responses. CpG ODNs, as immune modulators in radiotherapy, can potentially induce tumor cell necrosis and promote infiltration of inflammatory cells [41]. The study also found that the combination treatment enhanced the body’s defense against subsequent tumor attacks [42]. The preference for radiation enhancement in different types of cancer when the immune system is stimulated by CpG ODNs may be attributed to the tumor’s inherent characteristics or the type of immune response [41].

Bacterial OMVs are lipid bilayer vesicles produced by gram-negative bacteria, which can stimulate both the immune system and the bacterium itself [43]. Due to their immune-stimulating properties, low-dose radiation has been increasingly utilized in cancer treatment. In a study by the Patel group, they developed a multifaceted bacterial membrane-coated nanoparticle (BNP), consisting of a polyplex core coated with bacterial membrane and imide tissue, to combine the immunostimulatory capabilities of CpG ODN and bacterial membrane [44]. The polyplex core was composed of PC7A and CpG ODN. Combining BNP with radiation significantly enhanced the ability of immunologically “cold” tumors to activate the immune system, effectively serving as an in situ cancer vaccine [44]. Animal trials demonstrated promising results, highlighting the synergistic effects of TLR9 stimulation by CpG ODN and enhanced MHC I presentation by PC7A in improving the immune response to tumors. However, the precise relationship between bacteria, immunity, radiation, and tumors is not yet fully understood [44]. Therefore, further investigation is required to establish a theoretical framework for future studies.

Shiao and colleagues [45] emphasized the potential of modulating the gut microbiota to enhance the antitumor immune response to radiation treatment in breast cancer and melanoma. Commensal bacteria can influence the antitumor response by controlling immune cells that circulate within the gut immune compartment and indirectly through metabolites and the release of bacterial products [8, 46]. As highlighted by Shiao and colleagues [45], commensal fungi directly regulate intestinal mucosal immunity. They suggested that *Malassezia* spp. may be present in pancreatic tumors and could promote carcinogenesis. Shiao and colleagues [45] demonstrated how alterations in the gut microbiome could impact the anticancer immune response triggered by distant radiation, specifically by modulating intra-tumoral immunosuppression levels after radiotherapy in a Dectin-1-dependent manner. These findings support the notion that the microbiome can influence a tumor’s response to radiation, and both bacterial and fungal components of the gut microbiota can impact anticancer immunity.

According to research by Uribe-Herranz and colleagues [47], using a specific antibiotic, such as vancomycin, may enhance the cancer-fighting effects of radiation. Importantly, when vancomycin is taken orally, it remains localized in the gut, providing strong evidence that the observed phenotype results from local interactions between the immune response and the gut microbiota, which have long-lasting systemic effects [48]. Studies by Uribe-Herranz et al. [47] show that cytotoxic T cells and interferon-gamma (IFN-γ) are necessary for the synergistic treatment of radiation and vancomycin in improving antitumor properties. Gut microorganisms, particularly *Clostridia*, can metabolize dietary fiber in the colon, producing SCFAs such as butyrate, acetate, and propionate [49]. Studies have shown that SCFAs directly influence the functioning of DCs and macrophages. Propionate, for example, alters the biology of mouse macrophages and DCs in the bone marrow, impairing the ability of DCs to promote Th2 cell effector activity in the lungs [50]. Treatment of human DCs with butyrate and propionate dramatically reduced LPS-induced IL-6 mRNA and IL-12 gene expression while enhancing leukocyte trafficking. SCFAs also significantly reduced the production of several proinflammatory chemokines [51]. Similarly, according to Uribe-Herranz and colleagues [47], butyrate affected the expression of co-stimulatory molecules and antigen presentation by DCs but did not directly affect T cell IFN-γ production. However, research on how DCs directly influence antigen presentation during radiation is limited. In vivo administration of butyrate prevented the expansion of antigen-presenting cells (APCs) in tumor-draining lymph nodes (TDLNs) and reduced IFN-γ and IL-12 levels in the tumor.

Interestingly, mice treated with butyrate lost the additional anticancer benefits of vancomycin in the context of radiation. Furthermore, SCFAs produced by colonic *Clostridia* were found to induce the development of colonic Treg cells in mice [52]. In summary, these findings suggest that a complex interplay between microorganisms and immune system interactions mediates mice’s response to anticancer treatments. Based on the insights from these studies, researchers propose the use of personalized gut modifications to convert the local anticancer effects of radiation into a systemic response that can target metastatic diseases.

### Microbial metabolites can enhance the radiotherapy efficacy

A growing body of research suggests that microbial metabolites, such as SCFAs, may enhance the effectiveness of various cancer treatment approaches, including radiotherapy, chemotherapy, and immunotherapy [53]. SCFAs, particularly butyrate, have immunomodulatory properties that can influence anti-tumor responses by regulating the levels of immune-suppressing Tregs and tumor-killing CD4 + and CD8 + T cells [54]. Butyrate specifically inhibits histone deacetylases (HDACs), enzymes involved in cell cycle control and growth, and certain HDAC inhibitors have been explored as potential anti-cancer drugs [55]. Recent studies have shown that SCFAs such as butyrate can enhance the efficacy of radiotherapy by promoting anti-tumor immunity [53, 56]. Other microbial byproducts, such as indole compounds, have also demonstrated anti-tumor properties and may improve the effectiveness of radiotherapy [57].

A study by Park and colleagues [56] demonstrated that butyrate significantly enhances radiosensitivity in patient-derived organoids (PDOs) of colorectal cancer (CRC) by enhancing the transcriptional function of Forkhead box class O 3 A (FOXO3A) and inducing cell cycle arrest through the regulation of p21, p57, and GADD45. The study suggested that butyrate’s anticancer activity involves modulating HDAC-dependent transcriptional activity [58, 59]. While butyrate is typically used as an energy source in the normal colon, it accumulates in the nucleus of cancer cells, inhibiting their growth and inducing cell death, as cancer cells rely on glucose for energy [60–62]. Thus, targeting tumor metabolism, including the use of butyrate, may hold therapeutic potential [62]. Additionally, the study by Park et al. [56] found that non-responsive CRC-PDOs to butyrate and radiation combination therapy had lower levels of FOXO3A expression compared to responsive CRC-PDOs.

FOXO3A transcription factors play a crucial role in regulating cell growth, division, longevity, and the cell cycle [63]. Previous research has shown that Selumetinib (AZD6244) increases FOXO3A expression and inhibits the proliferation of colon cancer cells, and FOXO3A has also been found to increase cancer cells’ sensitivity to radiation [64, 65]. Thus, FOXO3A acts as a key modulator that enhances radiosensitivity. In CRC-PDOs, Park and colleagues [56]discovered that butyrate-induced inhibition of cell growth and cycling involved the FOXO3A-regulated genes p21, p57, and GADD45, which are known to suppress cancer cell proliferation [59, 62]. Moreover, GADD45 increases the radiosensitivity of cervical cancer cells by reducing the cytoplasmic localization of APE1 regulated by nitric oxide, while the inhibition of p57 by microRNA-221/222 may contribute to radioresistance [66, 67]. Butyrate increased radiosensitivity by blocking HDAC activity and modulating the FOXO3A/p21, p57, and GADD45 axis. Butyrate enhanced radiosensitivity through the Warburg effect, although only lactate concentrations in CRC-PDOs were investigated [53]. AMP-activated kinase (AMPK) inhibits tumor growth in vivo and regulates the Warburg effect in tumor cells [68]. Therefore, it is crucial to consider other signaling pathways, including the activation of AMPK. Recent studies have shown that FOXO3A inhibits glucose metabolism and tumor cell development in melanoma, and its expression is negatively associated with the expression of genes involved in glycolysis [69]. Furthermore, recent evidence suggests that glioblastoma cells may suppress the Warburg effect by activating the transcription of FOXO3A [70]. These findings collectively indicate a potential association between FOXO3A and the Warburg effect, emphasizing the need for further research in this area.

According to preliminary findings by Mete and colleagues [71], the administration of butyrate supplements in therapy demonstrates a higher level of efficacy. Patients treated with butyrate showed a complete response and a significantly better clinical condition compared to the control group and patients receiving continuous treatment. In the control group, 80% of patients showed no change in clinical status, whereas none of the patients receiving butyrate experienced this lack of improvement. Notably, the toxicity status decreased from moderate to mild, and full remission was observed in 10% of cases, which was not observed in the control group. Endoscopic examinations revealed improvement in 50% of patients receiving butyrate, whereas only 20% of the control group showed similar improvement. The lack of statistical significance in the latter findings may be attributed to the limited number of patients who have completed the ongoing trial thus far. While validation from a larger patient cohort is necessary, the findings gathered thus far hold promise. It is important to evaluate the effectiveness of this treatment in preventing radiation toxicity once the therapeutic efficiency of combining butyrate administration with standard therapy has been established.

In conclusion, emerging research indicates that microbial metabolites, specifically SCFAs, such as butyrate, can potentially enhance the effectiveness of various cancer treatments, including radiotherapy, chemotherapy, and immunotherapy. SCFAs, through their immunomodulatory properties, can influence anti-tumor responses by regulating immune-suppressing Tregs and tumor-killing T cells. Butyrate, in particular, inhibits HDACs involved in cell cycle control and growth, making it a promising target for anti-cancer therapies. Recent studies have demonstrated that SCFAs, including butyrate, can enhance the efficacy of radiotherapy by promoting anti-tumor immunity. Additionally, other microbial byproducts, such as indole compounds, have shown anti-tumor properties, suggesting their potential to improve the effectiveness of radiotherapy.

### Microbiota and radio-sensitization by improving tumor hypoxic microenvironment

The tumor microenvironment (TME) plays a crucial role in determining the efficacy of conventional radiotherapy [38]. The TME differs significantly from the normal internal microenvironment regarding tumor interstitial fluid pressure, hypoxia, acidity, and other physical and chemical characteristics. One of the significant challenges in clinical cancer therapy is the development of tumor radioresistance, which is influenced by factors such as increased expression of gene signaling pathways, hypoxia-inducible factor 1 (HIF-1), and vascular endothelial growth factor-A (VEGF-A) [72]. While hyperbaric oxygen treatment and radiation dose escalation have been explored as techniques to reduce the hypoxic microenvironment, their wide application in clinical practice is limited. Thus, innovative approaches are needed to improve the tumor hypoxic microenvironment. Certain facultative and anaerobic bacteria, including *Escherichia coli*, *Bifidobacterium*, and *Clostridium*, have been found to thrive in the hypoxic regions of tumors [73]. Two primary bacterial-based strategies are currently being investigated to increase oxygen levels in tumor tissues. These strategies involve delivering therapeutic agents to the tumor site to release oxygen or destroy oxygen-deficient tumor cells and enhance tumor circulation. The two strategies mentioned—bacteria and bacteria-derived membrane vesicles (MVs)—both leverage the unique properties of bacteria to improve drug delivery systems for cancer treatment [73]. Bacteria can be engineered to target tumor tissues, overcoming physical barriers that often impede traditional drug delivery methods. They accumulate in the tumor site and can stimulate antitumor immune responses, which helps in combating cancer more effectively [73]. Bacteria-derived MVs, on the other hand, are small particles released by bacteria that retain many of their beneficial properties. Like the bacteria themselves, these MVs can penetrate tumor tissues and deliver therapeutic agents directly to the cancer cells. They can also be modified genetically and chemically, allowing for the safe and efficient transport of anticancer drugs while minimizing damage to normal cells [73]. Both bacteria and their MVs show promising potential in delivering a variety of therapeutic agents, such as chemo-therapeutic, radio-therapeutic, photothermal-therapeutic, and immuno-therapeutic drugs, making them innovative tools in the fight against cancer [73].

In addition to *E. coli*, several bacterial strains can serve as effective delivery vehicles to transport specific carriers to tumor sites and target hypoxic cells, thereby improving the therapeutic efficacy of radiation [38]. Fu and colleagues [74] demonstrated that coupling *Bifidobacterium infantis* with a specific monoclonal antibody can effectively destroy the hypoxic tumor area, enhance radiation-induced DNA DSBs, and induce tumor cell death in a Lewis lung carcinoma (LLC) xenograft mice model. *B. infantis* is widely present in the GI tracts of humans and animals, showing no toxicity and helping to maintain a balanced bacterial population in the digestive system. The combination treatment of monoclonal antibody + *B. infantis* + radiation significantly reduced HIF-1α expression, substantially reducing tumor hypoxia and enhancing radiosensitivity [74]. Another approach to increasing oxygen supply is by improving tumor perfusion and oxygenation, as hypoxia at the tumor site is often caused by tumor vascular system damage and abnormalities [75]. Local treatment with Botulinum toxin-A has been reported to block the union of presynaptic membrane and vesicles, leading to increased tumor perfusion and oxygenation by inhibiting norepinephrine production at the neuromuscular junction. Notably, the dose used in this research is within the safe range for humans [76]. Fu and colleagues’ [70] study explored the mechanisms by which bacteria and their associated components can increase tumor radiosensitivity. In addition to reducing the tumor’s hypoxic microenvironment, two primary factors are considered: [1] the creation of an enzyme capable of infecting tumor cells and converting harmless prodrugs into radiosensitizers, and [2] the control of the cell cycle transition from a radio-resistant to a radiosensitive state. Previous research suggests that various naturally occurring bacterial components have the potential to directly enhance the effectiveness of tumor radiotherapy [77, 78]. One approach being investigated is suicide gene therapy, which involves the production of bacteria-derived prodrug-sensitive genes in tumor tissue. These genes target the conversion of non-toxic prodrugs into toxic metabolites that can destroy cancer cells. Bacterial toxins and the *cytosine deaminase (CD)* gene of *E. coli* are of particular interest in this context [79]. The *CD* gene can produce the CD enzyme, which catalyzes cytosine conversion into uracil. Radio-sensitization can be achieved by converting the prodrug 5-fluorocytosine to 5-fluorouracil (5-FU). Clinically, 5-FU is widely accepted as a radiation sensitizer for cancer treatment [80, 81]. In animal models, combination therapy involving intra-tumoral CD delivery, systemic administration of 5-FC, and radiation demonstrated significantly improved anti-tumor effects, even though in vitro studies did not clearly show a synergistic relationship between radiation and genetically designed molecular therapy. Similarly, when the *CD* gene was introduced into human colon cancer cells using a retrovirus vector, the prodrug administration, 5-FC, showed strong anti-cancer effects both in vitro and in vivo [79, 82]. Khil et al. [83]. confirmed that 5-FC selectively increased the radiation-induced cytotoxicity in colorectal carcinoma cells expressing the *CD* gene. Specifically, WiDr colorectal carcinoma cells transduced with the *CD* gene (WiDr-CD) showed significantly higher sensitivity to radiation compared to non-transduced parental WiDr cells when exposed to 20 micrograms/ml of 5-FC for 72 h prior to irradiation [83]. The sensitization enhancement ratio was 2.38, similar to that achieved with 5-FC. These results suggest that incorporating radiation therapy could greatly enhance the therapeutic efficacy of *CD* gene therapy for treating locally advanced colorectal carcinomas.

Furthermore, bacteria have the potential to alter the interaction between radiation and tumors, making them more sensitive to treatment and enhancing the therapeutic effects of radiotherapy. Microorganisms and their derivatives can reduce the hypoxic microenvironment by delivering therapeutic substances to the tumor site and indirectly or directly enhancing tumor perfusion. The development of bacterium-based nanomaterials has also attracted considerable interest as a means to enhance the use of bacteria in radiotherapy. Overall, microorganisms hold significant potential for enhancing the therapeutic effectiveness of radiotherapy, and further investigation is warranted to explore the unique properties of different bacterial products.

TME plays a crucial role in determining the success of radiotherapy. Strategies aimed at improving the TME and enhancing tumor radiosensitivity have been investigated. Bacteria, such as *E. coli*, *Bifidobacterium*, and *Clostridium*, thrive in the hypoxic regions of tumors and can be used as delivery vehicles to target and destroy hypoxic cells. Combining specific bacteria with monoclonal antibodies has shown promising results in reducing tumor hypoxia and increasing radiosensitivity. Additionally, approaches that improve tumor perfusion and oxygenation, such as local treatment with Botulinum toxin-A, have been explored. Furthermore, bacteria-derived enzymes and genes, such as CD, have been utilized in suicide gene therapy to convert prodrugs into radiosensitizers. These strategies have demonstrated improved anti-tumor effects in preclinical models. Overall, microorganisms and their derivatives have the potential to enhance the therapeutic effectiveness of radiotherapy by modifying the tumor microenvironment and targeting biological or physical factors that influence radiation sensitivity. Further research is needed to fully understand the unique properties of different bacterial products in this context.

## Relationship between the microbiome and radiotherapy-induced injury in various organs

Recent research has shed light on the potential involvement of the microbiota in both the development and prevention of radiation-induced damage in various organs [84]. One notable example is the impact of radiation on the gut microbiome, where radiation exposure can lead to changes in bacterial composition, with an increase in potentially harmful bacteria and a decrease in beneficial ones [84]. This alteration in the gut microbiota can trigger an inflammatory response and damage to the intestinal lining, resulting in symptoms such as diarrhea and abdominal discomfort. Similarly, radiation can also affect the skin’s microbiome, compromising its barrier function and increasing the susceptibility to infections. Furthermore, radiation exposure in the lungs can disrupt the lung microbiome, leading to inflammation and tissue damage, thereby making breathing more challenging [85]. This section explores the intricate relationship between the microbiome and radiation-induced injuries in specific organs.

### Skin

Dysbiosis of the skin microbiome has been implicated in various skin disorders and infections [86–88]. Disruptions to the stability of the skin microbiome can increase the risk of infection, as harmful bacteria such as *Staphylococcus aureus* can colonize the skin [89, 90]. The skin microbiome plays a crucial role in maintaining a diverse environment that helps protect against invading pathogens [91]. Understanding the mechanisms of cytokines that promote inflammation and characterizing the skin microbiome profiles associated with radiotherapy-induced dermatitis may provide insights into potential targets for reducing skin toxicities caused by radiotherapy. However, the connection between the immune system’s function and the skin microbiome remains poorly understood.

On the other hand, extensive research has been conducted on the association between atopic dermatitis (eczema) and the skin microbiota [92, 93]. In cancer patients, radiotherapy-induced dermatitis has been found to reduce bacterial diversity [94] significantly. Higher ratios of *Proteobacteria/Firmicutes* and dermotypes characterized by an abundance of *Pseudomonas*, *Staphylococcus*, and *Stenotrophomonas* have been associated with slower recovery or a higher propensity for persistent radiotherapy-induced dermatitis [94]. These findings should be further validated in cancer patients undergoing radiation, as the available data are insufficient to support them. Radiation-induced skin damage is a common side effect of radiation therapy in breast, lung, and colorectal cancer that significantly affects patients’ quality of life and presents challenges to healthcare providers [95–98]. Although the exact pathogenic mechanism of radiation-induced skin damage is unknown, it is often associated with disruptions to the structural integrity of skin microorganisms [99]. Analysis of clinical patients revealed that radiation significantly reduced microbial diversity in patients with chronic ulcers and slow-healing lesions, whose microbiome structure was severely compromised [96]. Similar findings have been observed regarding reducing microbial diversity in the GI tract following radiation [100]. The pathophysiological process of radiation-induced skin damage is primarily associated with immunological disorders, fibrosis, vascular injury, ROS damage, and epidermal abnormalities, resulting in reduced skin oxygen and nutrient supply [95]. These factors contribute to the decrease in microbial diversity caused by radiation.

Additionally, promoting the reestablishment of skin microbiota on wounds and activating the aryl hydrocarbon receptor (AhR) in keratinocytes can expedite the healing process of skin injuries [101, 102], highlighting the protective role of cutaneous bacteria in maintaining the integrity of the epidermis. A study carried out by Huang et al. [96]. exhibited the impact of radiation-induced skin damage on changes in the composition and function of cutaneous microbiomes, providing new insights into the potential mechanisms and microbial alterations involved in the development of radiation-induced skin damage. The prevalence of *Firmicutes* species, including *Lactobacillus*, *Lachnospiraceae*, *Streptococcaceae*, and *Staphylococcaceae*, appears to facilitate rapid healing in radiation-induced skin damage. Further research is needed to better understand the relationship between the skin microbiome and radiation-induced skin damage and to explore strategies for mitigating the detrimental effects of radiotherapy on the skin, such as the use of probiotics or topical prebiotics to promote a healthy microbiota or antibiotics to target harmful bacteria.

### Oral and mouth

Strong evidence indicates that radiation therapy has a significant impact on the oral microbiota, particularly by increasing the abundance of gram-negative bacteria such as *Klebsiella sp.* and *Pseudomonas aeruginosa*, *Candida albicans*, and certain gram-positive bacteria, notably *Lactobacillus sp.* [103]. While previous studies have shown a correlation between microorganisms found in the oral cavities of Head and Neck Cancer (HNC) patients after radiation and radiotherapy-induced toxicity, the specific actions and roles of the oral microbiota as explanatory mechanisms have not been thoroughly elucidated [103]. Stokman and colleagues [104] conducted a randomized clinical trial to evaluate the effects of polymyxin E, tobramycin, and amphotericin B, three topical broad-spectrum antibiotics, compared to placebo on the occurrence of radiation-induced oral mucositis in HNC patients. They assessed mucositis, changes in oral flora, quality of feeding, and changes in total body weight. The mucositis scores did not differ between the groups during the first 5 weeks of radiotherapy. The colonization index of *Candida* species and gram-negative bacilli was reduced in the polymyxin E 2 mg, tobramycin 1.8 mg, and amphotericin B 10 mg (PTA) group but not in the placebo group. No effect on other microorganisms was detected. In summary, selective oral flora elimination in patients undergoing head and neck irradiation does not prevent the development of severe mucositis.

Over the past decade, molecular analysis has identified several radiation-related alterations in the oral microbiota[100]. Following therapy, the overall bacterial count tends to decline and then gradually increase again, although the relative abundance of certain species and genera, such as *Bifidobacterium* and *Lactobacillus*, which are associated with obligate anaerobes in the gut, tends to increase [100, 105]. Regarding archaea, there appears to be minimal to no change due to radiation [100]. Furthermore, recent research has examined the effects of different radiation doses and found an inverse relationship between exposure and microbiome diversity [106, 107]. In Hu et al. [107]. study, four phyla (*Actinobacteria*,* Bacteroidetes*,* Firmicutes*, and *Proteobacteria*) and 11 genera (including *Streptococcus*, *Actinomyces*, and *Veillonella*) were consistently present, forming a core microbiome. Significant temporal variation in these core microbes’ relative abundance and a negative correlation between the number of operational taxonomic units (OTUs) and radiation dose were observed. These findings proposed a framework for defining a dynamic core microbiome under extreme conditions like radiotherapy, providing insights into predicting microbiome responses to ionizing radiation. Additionally, several recent studies have described alterations in the oral microbiota of individuals displaying tissue damage caused by radiation [108–110]. Other oral investigations have examined various radiation-induced alterations in the microbiome of supra-gingival plaque in HNC patients and potential correlations with the prevalence of dental caries [106, 111].

Investigations conducted after radiation administration have documented alterations in the relative abundance and diversity of the oral microbiome to understand better the etiology, incidence, and severity of oral mucositis. Zhu and colleagues [110], in their study on nasopharyngeal cancer (NPC) patients undergoing radiation alone or in combination with computed tomography (CT), found that increased richness of *Actinobacillus*, *Mannheimia*, *Streptobacillus*, unclassified *Pasteurellales*, and *Pasteurellaceae*, along with reduced bacterial diversity, were associated with higher severity of oral mucositis. However, certain gram-negative bacteria, such as *Fusobacterium* and *Haemophilus* were linked to vulnerability to oral mucositis, while others, including *Porphyromonas* and *Tannerella* were associated with increased severity of oral mucositis. Interestingly, a higher prevalence of *Candida* was neither related to the incidence nor the severity of oral mucositis [109]. A recent study found that oral mucositis usually starts 21 days after radiation therapy. It also found that different types of bacteria cause the condition to get worse at different times. For example, *Prevotella*, *Fusobacterium*, and *Streptococcus* were found just before oral mucositis, and *Megasphaera* and *Cardiobacterium* were found just before severe oral mucositis [108]. Numerous studies have examined changes in the dental plaque microbiome under various clinical conditions. It appears that radiation only induces transient changes in plaque composition. Previous research has investigated whether these radiation-induced alterations contribute to the development of dental caries. In a study conducted by Zhang et al. [111], a comparison was made between two cohorts of NPC patients (Twelve patients without radiation caries and nine patients with radiation caries, all following treatment for nasopharyngeal carcinoma) who underwent radiation therapy. The relationship between the oral microbiota and the presence or absence of carious lesions was examined. These researchers found that the oral microbiota after radiotherapy cannot explain the absence of radiation-induced caries in individuals. Another study identified a correlation between a reduced presence of *Abiotrophia*, a potentially protective oral gram-positive bacterium, and increased tooth decay [105]. Therefore, compared to oral mucositis, the changes in the oral microbiota induced by radiotherapy seem to play a smaller role or are not yet fully understood as explanatory mechanisms for the incidence of dental caries.

Early and ongoing preventive dental care is essential during and after radiation therapy, as the adverse effects of radiotherapy significantly diminish the overall quality of life for irradiated patients [112, 113]. In a study by Gaetti-Jardim et al. [114], the primary concern for patients was the occurrence of mucositis, which typically appeared within the first two weeks of radiation and was often accompanied by xerostomia (dry mouth) and candidiasis. More severe cases of mucositis were associated with oral candidiasis, poor hygiene, and colonization of the supra- and sub-gingival biofilms by members of the *Enterobacteriaceae* family and the *Candida* genus [114].

Changes in biofilm composition are mainly influenced by the severity and duration of xerostomia in the supragingival environment. According to an analysis by Schuurhuis and colleagues [115], individuals with less severe xerostomia resulting from radiation combined with oral preventive treatments to manage infection sites consistently showed reduced populations of major periodontal pathogens. The *Actinomyces*, *Capnocytophaga*, *Eikenella*, *Fusobacterium*, *Prevotella*, and *Porphyromonas* genera might have been more prevalent due to poor oral hygiene conditions and the development of gingivitis or periodontitis in almost every patient receiving radiotherapy, emphasizing the need for improved preventive measures [114]. Furthermore, mucositis-induced ulcerated lesions severely compromised oral hygiene in the majority of patients, even those who had received prior dental care, as the therapy primarily focused on extractions and dental restorations, which had a limited impact on the biofilm and hygiene conditions.

Xerostomia is commonly associated with the occurrence of severe gingival bleeding [116]. However, considering that individuals with mucositis and radiation exposure are more prone to developing periodontitis, further research is needed, particularly on patients who do not receive dental preventive treatment [117]. Vascular changes induced by radiotherapy, including reduced blood flow and potentially decreased redox potential of periodontal tissues, may contribute to increased populations of periodontitis-related gram-negative anaerobes in the sub-gingival biofilm. Nonetheless, the numbers of these anaerobes were reduced in the supra-gingival biofilm, which is more susceptible to the effects of xerostomia and changes in oral conditions among patients. This is likely associated with the increased acidity of the environment due to the proliferation of acidogenic cocci and a decrease in salivary buffer capacity in patients with xerostomia. Finally, it is important to note that the oral microbiome plays a critical role in maintaining dental health and regulating the immune system. However, radiation therapy has the potential to disrupt the balance of the oral microbiome, altering its microbial composition and increasing the risk of oral and mouth injuries. Further research is needed to develop more effective therapies for preserving the oral microbiome during radiation and better understand how the oral microbiota contributes to radiation-induced oral and mouth injuries.

### Lung

The lung microbiome comprises various microbes, including bacteria, fungi, and viruses, that play a crucial role in maintaining the respiratory system’s balance [118]. Microorganisms in the lungs protect against environmental harm, regulate the immune system, and sustain lung barrier function. Radiation exposure disrupts this balance, decreasing beneficial microbes and increasing harmful ones, leading to inflammation, lung tissue damage, and difficulty breathing [118].

Radiation-induced lung fibrosis typically becomes evident four weeks after exposure, while radiation-induced pneumonia may develop within hours, days, or weeks [119, 120]. Chen and colleagues [121] evaluated lung tissue structure, function, and inflammatory processes in experimental mice 21 days after irradiation. Their findings highlighted the impact of local chest radiation on the diversity of bacteria in the GI tract, thus influencing the gut-lung axis. Chen et al. [121] also analyzed the taxonomic ratios of intestinal bacteria and observed that FMT preserved the altered microbial composition caused by lung irradiation and mitigated radiation-induced lung damage. The documentation supported two main points: FMT effectively restructured the gut microbiota community, and this modification of gut flora influenced the oxidative stress and inflammatory state of lung tissues. It is worth noting that the detrimental effects of radiation exposure also include a decline in pulmonary function, which significantly impairs patients’ quality of life and increases inflammation and ROS production [121]. Chen et al. [121] further investigated the metabolome of gut bacteria in this context and specifically examined four target metabolites: trimethylamine N-oxide (TMAO), histidine hydrochloride hydrate, micronomicin, and prostaglandin F2α (PGF2α). PGF2α demonstrated the most pronounced protective effect on normal lung cells among these metabolites.

Oral replenishment of PGF2α in experimental mice increased its levels in fecal pellets, peripheral blood, and lung tissues, reducing lung inflammation and improving respiratory function post-irradiation. PGF2α activated the FP/MAPK/NF-κB axis, promoting cell proliferation and inhibiting apoptosis in radiation-challenged lung cells; silencing MAPK diminished PGF2α’s protective effects [121]. These findings support PGF2α as a key gut microbiota-produced metabolite and highlight a new avenue for treating radiotherapy-associated complications. Consequently, the presence of PGF leads to an increase in cell surface receptors containing PGF. When PGF is present, the coalescence of PI3K is reduced, resulting in the inhibition of relative signaling. In the study conducted by Chen et al. [121], it was observed that irradiation triggered the PI3K/AKT signaling pathway [121]. However, adding PGF2α hindered PI3K/AKT signaling due to its high affinity for the FP receptor. Previous studies have reported that PGF2α can activate MAPK signals, promoting the proliferation of endometrial cancer cells by binding to the FP receptor [122]. The MAPK superfamily mediates various signal transduction pathways, including extracellular signal-regulated kinase (ERK), c-Jun N-terminal kinase (JNK), and p38/MAPK, which are stimulated by ionizing radiation. Activation of MAPK/ERK signaling has been suggested as an effective regulator of cell proliferation, differentiation, and progression [123, 124]. Chen et al. [121] found that incorporating PGF2 increased the protein concentrations of p38, JNK, and ERK in healthy lung cells and tissues [121]. Cell nucleus size varies throughout the cell cycle, with the nuclear volume and the number of nuclear pore complexes quadrupling during interphase in dividing cells [125]. Chen and colleagues discovered that nuclear factor kappa B (NF-κB) was present in the cytoplasm regardless of radiation stimulation [121]. However, after PGF2 treatment, NF-κB became highly expressed and aggregated in the cell nucleus. Bioinformatics analysis also revealed a positive correlation between MAPK, a potential target gene of PGF2, and survival rates in lung cancer patients. The experimental findings were supported by the higher survival rates observed in patients with lung cancer tissue expressing high MAPK levels [121]. Therefore, MAPK/ERK1 may contribute to radioprotection and increase the chances of survival in lung cancer patients.

In a study by Li et al. [126], the radiation-induced changes in intestinal and pulmonary flora were examined. The findings demonstrated that the Phycocyanin (PC) intervention group had significantly lower whitening events than the irradiation group. Both PC pre-administration and medicinal management resulted in decreased concentrations of inflammatory mediators and LPS in lung tissue, serum, and the intestinal tract [126]. Although PC intervention significantly reduced flora diversity, chest irradiation caused imbalances in both lung and intestinal flora. Radiation-induced pulmonary fibrosis, a common and detrimental side effect of radiotherapy, negatively impacts patients’ quality of life and chances of survival [127]. Pulmonary fibrosis involves initial inflammatory processes and later fibrosis, which depend on the duration of irradiation [128]. Li et al. [126] employed hematoxylin and eosin (H&E) and Masson staining techniques to assess lung tissue fibrosis and injury levels. They observed varying degrees of alveolar injury and pulmonary fibrosis after irradiation of the chest cavity with a dosage of 20 Gy. The reduction of collagen fiber accumulation and inflammatory damage after both preventive and therapeutic PC administration suggests that PC has fibrosis-relieving properties. Comparing the compositional changes in lung flora with gut fauna, Li et al. [126] found numerous similarities between the two, whether evaluated at the phylum or genus level. For instance, the abundance of Firmicutes decreased following irradiation, while Bacteroidetes and Actinobacteria increased with PC administration [126].

Previous research has shown that, unlike *Bacteroidetes*, the abundance of *Firmicutes* is negatively associated with the levels of several inflammatory mediators [129]. This highlights the close connection between the lung and colon flora and the degree of inflammatory processes in lung tissue. Irradiation specifically reduced the quantity of *Lactobacillus*, *Lactococcus*, and *Bifidobacterium* at the genus level in the lungs and intestines [126]. However, following PC intervention, these three bacteria became more prevalent in gut and lung microbiota. Furthermore, both preventive and therapeutic administration of PC may reduce pulmonary fibrosis, although there are variations in controlling the composition of the flora, as indicated by the experimental findings of the PC + radiotherapy group and the radiotherapy + PC group. In conclusion, Li et al. [126]’ established a pulmonary fibrosis model in mice exposed to thoracic radiation and assessed PC’s protective and preventive effects. The results demonstrated that PC significantly reduced the levels of proinflammatory cytokines and LPS, suggesting a substantial reduction in radiation-induced pulmonary fibrosis. PC also decreased the levels of IL-6, TNF-α, and LPS in the lung, serum, and gut. It is evident that PC intervention can reverse the microflora abnormalities caused by irradiation. The relationship between pulmonary fibrosis-associated biochemical markers and the flora suggests the existence of communication between the colon and the lungs and that lung damage is directly linked to the composition of the flora.

### Intestine

Disruption of the gut microbiota caused by radiation can damage the lining of the GI tract, causing symptoms such as diarrhea, abdominal discomfort, and an inflammatory response [130]. The condition known as “radiation-induced enteritis” refers to inflammation of the intestinal mucosa caused by free radicals generated through ionization [131]. The characteristics of radiation enteritis include a compromised intestinal mucosal barrier, heightened levels of inflammatory mediators, increased invasion of pathogens and release of endotoxins, and a weakened immune defense[132, 133]. Reports indicate that enteritis related to radiotherapy often leads to reductions in the radiation dosage. Such reductions can significantly impair bodily functions and increase the mortality rate among cancer patients [134, 135]. High doses of radiation can cause shrinkage of intestinal villi, injury to the intestinal epithelium, elevated apoptosis, and increased inflammatory responses. Moreover, radiation can disrupt the integrity of the intestinal epithelial barrier, resulting in increased intestinal permeability, diarrhea, and disturbances in water and electrolyte digestion. Radiation enteritis can be categorized into five stages: the initial phase, where ROS causes DNA damage; the primary damage reaction phase, characterized by inflammation and apoptosis; the signal amplifying phase, where further inflammatory processes and apoptosis occur; the ulceration phase, marked by disruption of the epithelial barrier and promotion of bacterial translocation; and the rehabilitation phase, where cell growth takes place after radiotherapy has ceased [136].

Segers and colleagues [137] aimed to investigate radiation-induced mucositis in the intestine, a common clinical side effect of pelvic radiotherapy, and conducted a comprehensive analysis of the microbiota response. The chronological succession of biological processes, as documented by Cinausero et al. [138], can be succinctly outlined as follows: The administration of pelvic irradiation elicited a primary reaction primarily in the ileum, with a secondary effect observed in the colon. This response was characterized by the occurrence of apoptosis and inflammatory processes, as evidenced by histological analysis and the measurement of myeloperoxidase activity [137]. Subsequently, as these destructive signals propagated, the epithelial barrier broke down due to the loss of tight junctions, facilitating the dissemination of bacteria into the mesenteric lymph nodes. After the cessation of irradiation, increased Ki67-mediated cell growth ultimately activated healing mechanisms [137]. Notably, the study also revealed that the functional and structural changes in the irradiated gut were associated with dysbiosis. Regarding compositional changes, the analysis of relevant OTUs affected by pelvic irradiation revealed distinctive shifts, with members of the *Ruminococcaceae* family, known to increase in mice after repeated irradiations, being particularly emphasized. This family has been reported to be radiation-resistant. The *Lachnospiraceae* and *Clostridiaceae* families showed significant changes after sub-lethal exposure in mice [139, 140]. Different responses to irradiation were observed among members of the *Lachnospiraceae* family, which aligns with reported cases of intestinal damage [141–143]. Significant increases were observed in OTUs associated with the genera *Anaerotruncus*, *Oscillibacter*, and *Clostridium* cluster XIVb within the *Ruminococcaceae* and *Lachnospiraceae* families, consistent with findings in irradiated mice and minipigs [142, 144]. Notably, the number of *Oscillibacter* species was positively correlated with radiation intensity in larger animals. *Oscillibacter* and *Anaerotruncus* species were found in higher numbers in mice and individuals with inflamed and hyper-permeable intestines [145, 146]. Conversely, as previously demonstrated in mice exposed to radiation and mice with inflammatory bowel disease (IBD), the proportions of OTUs from the *Porphyromonadaceae* family were significantly reduced [137]. These identified biomarkers could potentially be utilized for disease identification, prognosis, and the development of personalized treatments for radiation-induced intestinal problems. In summary, utilizing a multi-level approach, Segers and colleagues [137] demonstrated rapid crypt epithelial cell death, an inflammatory response, compromised barrier integrity, and translocation of intraluminal bacteria into mesenteric lymph nodes following acute pelvic irradiation. The dysbiosis indicators specific to pelvic irradiation, such as the *Ruminococcaceae*, *Lachnospiraceae*, and *Porphyromonadaceae* families, had a prolonged but significant impact on the beta diversity of the gut microbiota after radiation-induced GI mucositis [137]. Further research is necessary to develop improved interventions for maintaining the gut microbiota during radiation and to better understand the mechanisms by which the gut microbiome contributes to radiation-induced intestinal harm.

### Brain

Recent investigations have shed light on the crucial role of the gut-brain axis, which represents the bidirectional communication between the gut microbiome and the brain, in the development and progression of brain injury following radiation therapy, despite limited research on the microbiome’s relationship with radiation-induced brain damage [147]. The gut microbiota can communicate with the brain and spinal cord through various pathways, such as the vagus nerve and the immune system [148]. Radiation exposure can alter the composition of the gut microbiota and increase the risk of inflammatory responses that can impact the brain [147]. Moreover, radiation may also damage the BBB, which regulates the exchange of nutrients and waste products between the blood and the brain, leading to a higher susceptibility to infection and inflammatory processes within the brain.

The impact of radiation-induced neurotoxicity via the gut microbiota axis on the quality of life of radiotherapy patients remains poorly understood. Venkidesh and colleagues [149] conducted a study using gut microbiome *16 S rRNA* sequencing, which revealed that pelvic radiation at a dose of 6 Gy resulted in alterations in the gut bacterial composition. They observed a significant increase in the abundance of specific bacterial genera, including *Parabacteroides*, *Sutterella*, *Desulfovibrio*, *Ruminococcus*, *Treponema*, *Alistipes*, *Parasutterella*, *Helicobacter*, *Eubacterium*, and *Tyzzerella*, in samples collected on day 12 after treatment with 6 Gy of pelvic irradiation. These increased abundances of bacterial genera may serve as indicators of neurotoxicity [149]. In addition to its role in facilitating beneficial communication within the host, the gut microbiota also influences the host’s development, well-being, and susceptibility to diseases. DNA damage is a well-studied biological mechanism underlying radiation-induced harm, as it disrupts various signaling pathways critical for the cell cycle, apoptosis, and stress response [150]. After radiation therapy to the head region of the rats, the hippocampus is particularly vulnerable to the effects of ionizing radiation, and previous studies have shown that radiation-induced impairments in learning and memory are associated with increased apoptosis and decreased neurogenesis in the hippocampus [151]. This vulnerability may stem from cellular components involved in both neurogenesis and cell death within the hippocampus [152]. Venkidesh et al. [149] conducted a study that revealed that pelvic radiation has the potential to induce substantial neuronal cell death in the dentate gyrus (DG) and Cornu Ammonis 2 (CA2) regions of the hippocampus. This finding supports the notion that radiation can indirectly affect memory and cognition. Furthermore, the investigation focused on plasma glial fibrillary acidic protein (GFAP), a specific marker for astrocytes that plays a crucial role in the cytoskeleton. The degree of GFAP expression can indicate astrocyte damage, as active astrocytes exhibit increased GFAP levels [153]. The presence of elevated levels of reactive astrocytes in the hippocampus’s CA1, CA2, and DG regions suggests the possibility of astrocytosis and neuroinflammation, indicating increased activation of astrocytes in the hippocampus. Following radiation exposure, the brain’s neuronal, glial, and vascular components may undergo various molecular, cellular, and functional changes depending on the severity of the exposure. The hippocampus has been the focus of much research due to its critical role in memory and adult neurogenesis. Venkidesh et al. [149] found that pelvic radiation could significantly decrease the number of mature neurons in the hippocampus; however, further research is needed to confirm the contribution of gut dysbiosis to reduced neuronal maturation, impaired memory, and neurotoxicity. Nonetheless, studies on gut dysbiosis have shown an increase in specific bacterial genera such as *Parabacteroides*, *Bacteroides*, *Clostridium*, *Sutterella*, and *Treponema*, suggesting their potential involvement in altered brain function, as observed in the current study. According to Cryan et al. [154], the brain can trigger signaling pathways that affect immune and metabolic activity as well as host behavior, while the gut microbiota can influence the functioning of the central nervous system (CNS) by modulating behavior, memory, and cognition. Venkidesh et al. [149] found that radiation-induced bacterial dysbiosis significantly reduced exploratory behavior in rats. Consistent with Venkidesh et al. [149], pelvic radiation-induced gut dysbiosis may lead to decreased BDNF levels and significantly reduced NMDA expression. In summary, these findings demonstrate that a single dose of 6 Gy pelvic radiation in a rat model caused significant damage to intestinal tissue and resulted in distinct and significant alterations in the gut microbiota. Surprisingly, these findings support the hypothesis that non-targeted radiation effects may result in significant losses in neuronal survival, development, and exploratory behavior, as well as lower expression of genes involved in brain plasticity. Further studies are needed to develop more effective strategies for promoting the gut-brain axis during radiation therapy and to gain a better understanding of the mechanisms through which the gut microbiota influences brain damage.

## Microbiome-based therapies against radiotherapy-induced injury

**Fig. 2:**
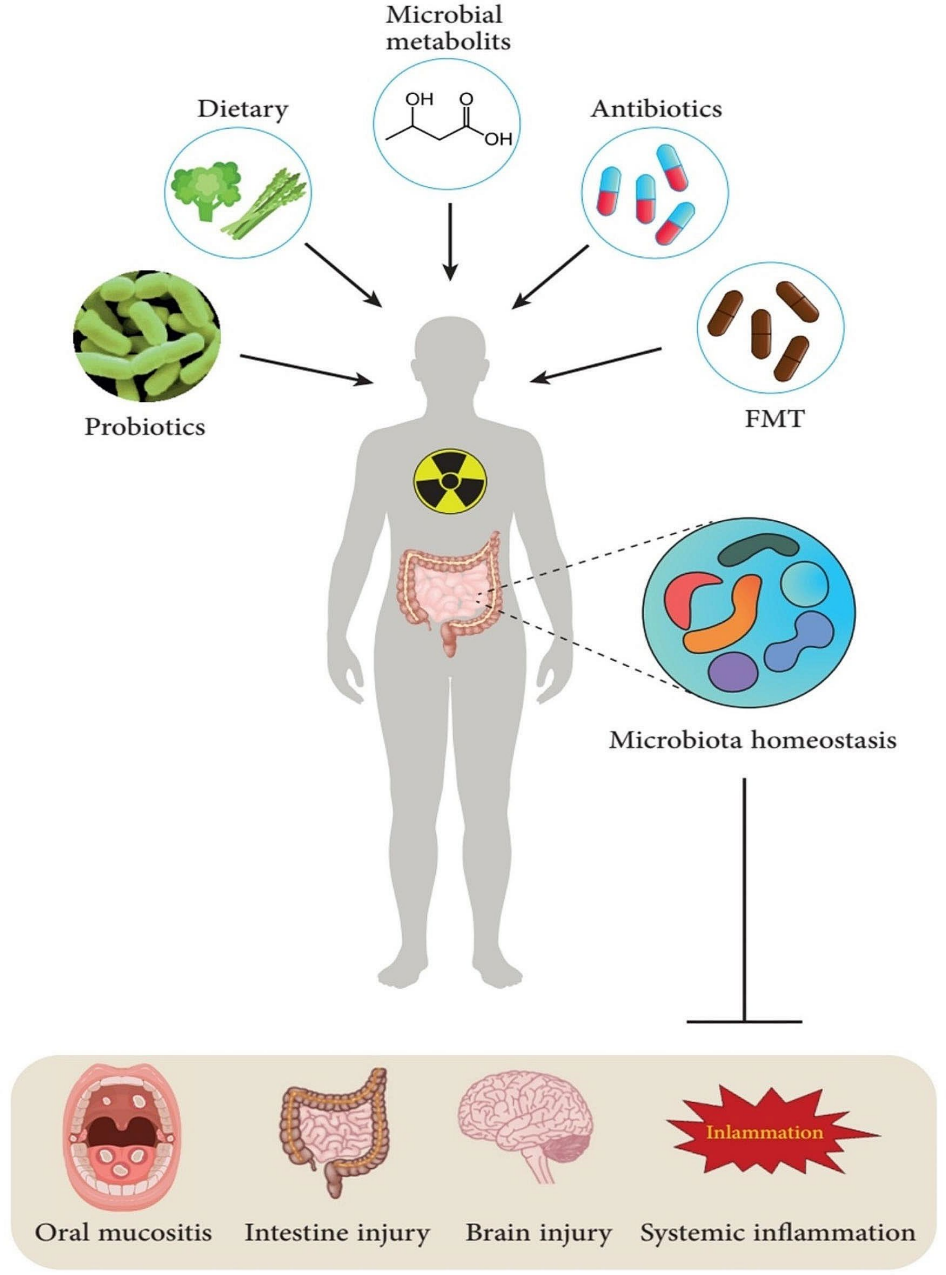
Microbiome against radiotherapy-induced injury. Overview of various microbiome-based strategies that can be used to reduce radiotherapy-induced injury on the human body. The figure is divided into five sections, each discussing a different approach to reducing. The first section discusses the use of probiotics to combat radiotherapy injury. Studies have shown that oral probiotics can reduce the incidence of oral mucositis and improve immunity in patients undergoing concurrent chemoradiotherapy. Probiotics have also been found to reduce the use of antidiarrheal medication and the incidence of abdominal pain in patients receiving radiotherapy. The second section discusses dietary interventions that can be used to reduce radiotherapy-induced injury. Guiqi Baizhu decoction and Spirulina platensis have been shown to have anti-inflammatory and antioxidative effects, respectively, and can regulate the gut microbiota, making them beneficial for preventing and treating intestinal diseases. Similarly, dietary interventions and microbiota-based strategies have been studied for their potential to reduce the adverse effects of radiation therapy on the brain. The third section focuses on microbial metabolites that can reduce radiotherapy-induced injury. Gut microbiota-derived metabolites, such as 3-hydroxybutyrate and short-chain fatty acids, have been shown to play a radioprotective role and can inhibit the expression of proinflammatory cytokines and exert anti-tumor activity. Dietary pectin and soluble dietary fiber may reduce radiation-induced EMT and intestinal fibrosis by regulating intestinal flora and SCFA concentration. The fourth section discusses the use of antibiotics to mitigate radiotherapy-induced injury. Antibiotics pretreatment can improve the viability of mice with postradiation intestinal damage by regulating the LPS/TLR4/MyD88/NF-κB p65/macrophage polarization/TGF-β1/Smad-3 signaling pathway. Antibiotics can also enhance the reconstitution ability of intestinal microbiota after radiation and reduce intestinal wall fibrosis by downregulating TGF-β1/Smad-3 signaling pathways in radiated mice. Finally, the fifth section discusses fecal microbiota transplantation (FMT) as a potential strategy to reduce radiotherapy-induced injury. FMT can increase the level of microbial-derived indole 3-propionic acid and reduce inflammation in the gut, potentially reducing the severity of radiotherapy-induced gastrointestinal (GI) toxicity. Overall, these strategies provide promising avenues for reducing the negative effects of radiotherapy and improving patient outcomes

Microbiome-based treatments have shown promise in mitigating the side effects of radiation therapy-induced damage in various parts of the body (Table 3) [155]. These treatments aim to restore balance to the microbiome and promote the growth of beneficial bacteria while suppressing harmful ones. Several microbiome-based treatments, including probiotics, dietary modifications, metabolites, antibiotics, and FMT, are currently being explored. The following sections have reviewed and discussed these treatments (Fig. 2).

**Table 3:** Effect of the microbiome on reducing the radiotherapy-induced injury

Condition	Radiotherapy	Study setting	Microbiome derived approach	Toxicity type	Mechanism	Outcome	Ref.
Gynecologic cancer	1.8 to 2.0 Gy	Clinical Trial	Probiotics	Abdominal pain and defecation urgency	-	Consumption of the probiotics decreased the intensity of the complaints of grinding abdominal discomfort and defecation urgency as well as the proportion of days with these symptoms in contrast to the placebo.	[276]
Sigmoid, rectal, and cervical cancer	-	Clinical Trial	Probiotics	Enteritis and colitis	-	In comparison to VSL#3 users, more people receiving placebos had radiation-induced diarrhea, and more placebo patients experienced grade 3 or 4 diarrhea.	[277]
-	4 Gy	In vivo	Probiotics	Bloody diarrhea, gastritis, inflammation, and breakdown of the gut–blood barrier	-	Probiotic therapy greatly reduced the villi height and mucosal thickness injuries caused by radiation. Additionally, probiotics decreased the neuronal inflammation caused by radiation in the brain’s cortex, CA2, and DG area.	[278]
-	12 Gy	In vivo	Probiotics	Apoptosis and crypt survival	Toll-like receptor (TLR) -2/cyclo-oxygenase-2	*Lactobacillus rhamnosus* GG (LGG) improved crypt survival and decreased radiation-induced epithelial damage. Constitutively cyclooxygenase-2 (COX-2)-expressing mesenchymal stem cells were moved to the crypt base via a TLR-2/MyD88 signaling pathway.	[170]
-	11 Gy	In vivo	Probiotics	Diarrhea and inflammation	Changed fecal flora	According to this research, taking a probiotic altered the fecal flora and shielded the gastrointestinal (GI) tract against the danger of radiation-induced diarrhea.	[279]
Nasopharyngeal Carcinoma	60 Gy	Clinical Trial	Probiotics	Oral mucositis	CD4 + T cells, CD8 + T cells and CD3 + T cells	This research discovered that altering the gut microbiome decreased oral mucositis severity and improved patient immune responses.	[280]
Pelvic cancer	1.8 Gy	Clinical Trial	Probiotics and honey	Diarrhea	-	As a consequence of using probiotics or probiotics combined with honey, patients’ daily bowel movements, the severity of their diarrhea, and their need for antidiarrheal medicine all decreased.	[281]
Gynecological cancer	1.8 and 52.2 Gy	Clinical Trial	Inulin and fructo-oligosaccharide	Enteritis	-	Prebiotics enhanced the consistency of their feces in individuals with gynecologic cancer receiving radiation.	[176]
Gynecological cancer	52.2 Gy	Clinical Trial	Inulin and fructo-oligosaccharide	Enteritis	Proliferation of Lactobacillus and Bifidobacterium	After radiation, *Lactobacillus* and *Bifidobacterium* were able to recover thanks to the prebiotic mixture’s stimulation of bacterial reactivation.	[178]
-	0.88, 4, 7 and 12 Gy	In vivo	High-fat diet (HFD)	Marrow and GI toxicity	Altered bacterial structure	Exclusively in irradiated males, does simvastatin oral gavage enhance GI tract performance and epithelial integrity and reduce hematopoietic system harm, while only in irradiated females does feeding with HFD overtly reduce bone marrow and GI toxicity.	[282]
-	3 Gy	In vivo	Antibiotics	-	Regulating gut microbiota	When antibiotics were supplied before and after total body irradiation (TBI), they affected the bacteria diversity in the stomach and decreased longevity.	[283]
Normal	2 Gy and 10 Gy	In vivo	Antibiotic cocktail (Abx)	Inflammation, fibrosis in the intestine	TLR4/MyD88/NF-κB signaling	By lowering inflammatory processes and avoiding intestinal fibrosis, abx pretreatment greatly increased the survival rate of mice following radiation therapy and reduced intestinal damage.	[242]
Normal	15 Gy	In vivo	Quercetin inclusion complex gels (QICG)	Brain injury	Regulating gut microbiota	After oral QICG delivery, the relative variety and abundance of the gut microbiota in RIBI mice altered. Additionally, the amount of anxiety was reduced while spontaneous behavior, short-term memory, and capacity were all enhanced. Last but not least, alterations in physical symptoms were seen, along with a drop in Tumor necrosis factor alpha (TNF-α) and IL-6 levels.	[219]
-	6 Gy	In vivo	Guiqi Baizhu decoction (GQBZD)	Enteritis	Microbiota dysbiosis	In rats given X-ray radiation treatment, GQBZD decreased body weights, water intake, food intake, the amount of diarrhea, the quality-of-life score, inflammatory responses, and immune function.	[284]
Normal lung	20 Gy	In vivo	Phycocyanin	Pulmonary fibrosis	Regulation of the lung and gut microbiota composition	Radiation-induced lung damage was decreased by phytocyanin, and inflammatory mediators were present at lower levels.	[126]
Abdominal or pelvic solid tumors	8.5, 12 and 16 Gy	In vitro and in vivo	Microalgae-based oral microcarriers	Intestinal injury	-	Spirulina platensis, a natural microcarrier, dramatically increased the radioprotection of Amifostine on the entire gut by taking advantage of the extensive intestinal biodistribution.	[215]
Xenograft tumor	4 Gy and 7.2 Gy	In vivo	Indole 3-propionic acid (IPA)	Inflammatory mediators such as IL-6 and TNF-ɑ in the intestine	acyl-CoA	In particular, after irradiation, mice treated with IPA showed reduced systemic inflammatory levels, recovered hematogenic organs, catabatic myelosuppression, better GI function, and epithelial integrity.	[285]
Rectal cancer	25 Gy	In vivo	*Akkermansia* *muciniphila*	Proctopathy	G-protein-coupled receptor 43 (GPR43)-mediated IL-6 signaling	Administration of *A. muciniphila* dramatically raises the concentration of 3-hydroxybutyrate (3HB), reduces the expression of IL-6 and GPR43, and lessens the severity of radiation damage in mice.	[286]
Nasopharyngeal carcinom	2, 10, 20, 30, 40, 50, 60 and 70 Gy	In vitro and clinical	Oral microbiota	Oral mucositis	Bacterial dysbiosis	In individuals with nasopharyngeal cancer, alterations in the oral microbiota were associated with the development and exacerbation of radiotherapy-induced mucositis.	[110]
Nasal, oral and laryngeal cancer(NOALC)	30 Gy	In vivo	Oral Microbiota	Oral mucositis	Oral and intestinal bacteria taxonomic proportions	Upon local head and neck radiation, oral microbiota transplantation prevented weight loss, decreased systemic and glossal inflammatory conditions, and reorganized the tongue’s dramatic disarray.	[287]
Lung tumor	0.8 and 15 Gy	In vitro and in vivo	Faecal microbiota transplantation (FMT)	Pneumonia	prostaglandin F2α (PGF2α) via Mitogen-activated protein kinase (MAPK) / nuclear factor-κB (NF-κB)	With radiation exposure, PGF2α activated the FP/MAPK/NF-κB axis to increase cell proliferation and prevent apoptosis; MAPK silencing reduced PGF2α’s beneficial impact on radiation-challenged lung cells.	[288]
-	8.2 to 9.2 Gy	In vivo	*Lachnospiraceae* and *Enterococcaceae*	DNA damage	Promoting hematopoiesis and attenuating GI damage	Mice were radiation-resistant thanks to short-chain fatty acids (SCFAs), particularly propionate, which mitigate DNA damage and reactive oxygen species production in hematological and GI problems. Kynurenic acid (KYNA) and 1 H-indole-3-carboxaldehyde (I3A), metabolites of the tryptophan pathway, furthermore provide long-term radioprotection.	[289]

### Probiotics against radiotherapy injury

Probiotics are living bacteria that, when consumed in sufficient quantities, promote the health of their host by influencing the processing of food and energy by the commensal microbiota [156]. They can be obtained through supplements or certain fermented foods. Probiotics typically consist of complex mixtures of microorganisms, often including bacteria from the *Lactobacillus* and *Bifidobacterium* genera, and they function in specific ways [157]. Numerous studies have examined the potential of probiotics in reducing radiation-induced damage. For example, a comprehensive review and meta-analysis of randomized controlled trials revealed that probiotics reduced the risk of radiation-induced diarrhea by approximately 40% [158]. Although the exact mechanisms through which probiotics may protect against radiation-induced damage are not fully understood, they are believed to involve various factors, such as reducing inflammation, enhancing the function of the gut barrier, and modifying the gut microbiota.

#### Probiotics against oral mucositis

The probiotic cocktail considerably decreased oral mucositis, as reported by Jiang et al., due to a dramatic improvement in the patient’s immunological response [159]. The results confirmed the preventive impact of the probiotic combination (Bifico, SHANGHAI SINE PHARMACEUTICAL CO.LTD, SFDA approval number: S10950032) contained *Bifidobacterium longum*, *Lactobacillus lactis*, and *Enterococcus faecium*) against oral mucositis, as only 15.52% of individuals in the concomitant chemo-radiotherapy (CCRT-P) group experienced grade 3 oral mucositis, compared to 45.71% in the CCRT group [159]. The tumor response to CCRT was not affected by the probiotic cocktail, as all recruited patients had similar objective response rates. This outcome aligns with a previous study that demonstrated how a specific probiotic, *Lactobacillus brevis* CD2, reduced the occurrence of radiochemotherapy-induced oral mucositis (grades 3 and 4) in individuals with head and neck squamous cell carcinoma undergoing chemoradiation treatment [160].

Radiation damage to the oral mucosa can cause various symptoms, including changes in saliva quality [161]. Due to radiation therapy-induced damage to the salivary glands, large salivary glycoproteins (e.g., immunoglobulin A (IgA)) that coat the oral mucosa surface have limited ability to bind together. Glycoproteins act as a barrier for oral cavity surface cells and reduce the adhesion of bacteria to the oral mucosa. In comparison to B-CCRT-P (before the treatment of radiotherapy plus chemotherapy plus the probiotic combination) patients who received equivalent radiation doses to the oral cavity, left/right parotid gland, and left/right submandibular gland, the administration of the probiotic cocktail significantly increased T-cell counts. Since probiotics have demonstrated their ability to modulate human immune defenses against pathogens and tumor cells and are essential for overall immune system responses, the increased numbers of CD3 + T cells, CD8 + T cells, and CD4 + T cells in the A-CCRT-P group underscore their importance [11, 39]. Additionally, blood tests were conducted to assess patients’ health in the A-CCRT (after treatment with radiotherapy plus chemotherapy plus a placebo) and A-CCRT-P (after treatment with radiotherapy plus chemotherapy plus the probiotic combination) groups. The probiotic cocktail significantly restored CD3 + T, CD4 + T, CD8 + T cells, hemoglobin levels, and the lymphocyte ratio to normal levels.

In light of the restricted sample size and the inherent variability of the oral microbiota, Jiang et al. [159] employed high-throughput sequencing techniques to monitor the dynamic fluctuations in the gut microbiota, as opposed to previous studies that focused on the oral microbiota. Their findings revealed that *Firmicutes*, *Bacteroidetes*, *Proteobacteria*, and *Actinobacteria* were the dominant phyla and accounted for the majority of the sequencing data in the healthy people (HP), B-CCRT, A-CCRT, B-CCRT, and A-CCRT-P groups. *Firmicutes* have been associated with energy resorption and may play a role in developing diabetes and obesity. Previous research has shown that *Firmicutes* constitute the majority of the gut microbiota in mice and humans [162, 163]. The presence of *Bacteroidetes* in the human digestive system is strongly linked to dietary modifications and adverse effects during cancer treatment [164]. According to the main coordinate analysis results presented by Jiang et al. [159], CCRT treatment significantly disrupted the GI diversity of the patients. Specimens from the A-CCRT group were distinctly separated from specimens in the HP and B-CCRT groups. However, administering the probiotic mixture (containing *B. longum*, *L. lactis*, and *E. faecium*) in the A-CCRT-P group substantially improved microbial diversity compared to the HP and B-CCRT-P groups within the CCRT group. This finding indicates that the probiotic combination effectively enhanced efficacy, reduced radiation toxicity, and preserved a balanced bacterial composition in the gut. Importantly, the probiotic cocktail used in this study was well tolerated and did not cause an increase in adverse events. By improving radiochemotherapy-induced microbial disturbances in the gut, the probiotic cocktail demonstrated the potential to enhance absorption, digestion, energy generation, and immunity while possibly alleviating oral mucositis in nasopharyngeal cancer patients. These results provide the first evidence that the probiotic cocktail significantly enhances patient immunity, reduces oral mucositis, and promotes the restoration of microbial diversity after CCRT. Therefore, based on the findings of this randomized clinical investigation, oral probiotics have shown promise in preventing oral mucositis in cancer patients undergoing radiochemotherapy.

#### Probiotics against intestinal adverse injury

Probiotics have emerged as a promising approach for mitigating the GI side effects induced by cancer treatments, specifically radiation. In a study by [165], patients who received *Lactobacillus acidophilus LA-5* and *Bifidobacterium animalis subsp. lactis BB-12* experienced significantly reduced usage of the antidiarrheal drug loperamide. They also reported a decrease in grade 2 stomach discomfort and episodes of abdominal pain in the days following radiation. Another study involving probiotics and prebiotics supplementation (*Bifidobacteria* and *E. coli*) demonstrated a reduction in fecal calprotectin levels, as well as a decrease in the frequency and severity of vomiting during a 7-week period of chemotherapy and radiation treatment [166]. The use of the probiotic supplement VSL was found to decrease the frequency of radiation-induced diarrhea and the number of daily bowel movements [167]. In women with gynecological malignancies undergoing radiotherapy, fermented milk containing live *L. acidophilus* bacteria was shown to reduce radiation-induced diarrhea [168]. Additionally, individuals taking *Lactobacillus rhamnosus* supplements exhibited reduced radiation-induced digestive damage, improved fecal consistency, and decreased bowel movements [169].

In mice, prior administration of *Lactobacillus* spp. probiotics to the small intestine resulted in reduced epithelial apoptosis and enhanced crypt survival after irradiation. A study by Ciorba et al. [170] demonstrated that gavage with *Lactobacillus* spp. or LGG (*Lactobacillus* GG)-CM conditioned medium) provided significant benefits, with LGG offering the highest level of protection. However, administration of *Bifidobacterium* sp. did not have any radioprotective effects. The intact signaling of TLR-2, Myeloid differentiation factor 88 (MYD88), and Cyclooxygenase (COX)-2 were found to be essential for the radioprotective effects mediated by LGG. Importantly, LGG administration before radiation, but not after, was shown to be protective, indicating that its effects are primarily preventative rather than curative [170].TLR-2 ligands, such as peptidoglycan and lipoteichoic acid, are present in the cell walls of gram-positive bacteria, including LGG [170]. The study by Ciorba et al. [170] suggested that LGG peptidoglycan or lipoteichoic acid might be released into the medium and interact with TLR-2, as LGG-CM also exhibited radioprotective properties. This finding marked the first instance where TLR-2 was implicated in mediating a probiotic-based biological impact. Furthermore, the anti-inflammatory effects of VSL#3, a combination of different bacteria, were found to be mediated through TLR-9 signaling, representing the only known example to date of therapeutic probiotic effects mediated by TLR signaling [171]. ROS, AKT stimulation, and heat shock proteins have all been associated with the biological effects of LGG [172–174], although it remains unclear whether TLR-2 activation plays a role in mediating these effects. It is likely that additional TLRs also play a role in the host’s response to radiation given the variations in radiation-induced apoptosis between *TLR-2-/-* and *MyD88-/-* mice.

COX-2 mediated the radioprotective effects of LGG in the study conducted by Ciorba et al. [170, 175]. Intestinal PGE2, which can be produced by either COX-1 or COX-2, has been shown to have radioprotective properties [175]. The radioprotective activities of LPS via TLR-4 are dependent on COX-2 and PGE2 [231]. Administration of LPS parenterally leads to increased COX-2 expression in the gut. Although LGG also operates through a COX-2-dependent mechanism, its gavage did not impact the expression of COX-2 or the concentration of PGE2 in the small intestine. The overall number of COX-2-expressing cells in the gut was unaffected by LGG; however, the number of COX-2-expressing cells near the crypts increased in response to LGG. These migratory cells express surface proteins associated with mesenchymal stem cells (MSCs) and COX-2. Only intestinal and colonic MSCs constitutively produce high levels of COX-2, while bone marrow-derived MSCs have low COX-2 expression [170]. The elevated COX-2 expression in colonic MSCs depends on fibroblast growth factor 9 (FGF9) produced by epithelial cells rather than TLR signaling. The migration of intestinal MSCs observed in the study was found to be TLR-2 specific, although the precise impact of TLR-2 signaling on their migration remains unclear. The functional expression of TLR-2 in MSCs suggests that TLR-2 signaling likely occurs in these cells. It is also possible that the TLR-2 ligand binds to a cell in or near the crypt, which then secretes a chemotactic protein that promotes MSC migration toward the crypt. These COX-2-expressing MSCs would then work to protect adjacent epithelial stem cells from radiation-induced death by producing PGE2 [170]. Given that PGE2 has limited stability and operates over short distances, the PGE2-producing MSCs and the epithelial stem cells must be physically adjacent to each other. Ciorba et al. [170] demonstrated that TLR-dependent migration of COX-2-positive MSCs was associated with dextran sulfate sodium (DSS)-colitis and provided radioprotection in the small intestine. This suggests that TLR-dependent migration of COX-2-expressing MSCs may represent a comprehensive mechanism for preserving colon and intestinal epithelium.

In a study by Garcia-Peris et al. [176], it has been explored the potential benefits of using pharmacologic doses of two prebiotics (inulin and fructo-oligosaccharide) in women with gynecologic cancer undergoing abdominal radiation treatment. The aim was to improve their quality of life and reduce the incidence of acute radiation enteritis (ARE). Rectal biopsies taken from individuals receiving pelvic radiation have revealed stromal inflammation, acute cryptitis, crypt abscesses, and surface epithelial atrophy [177, 178]. It has been observed that abdominal irradiation in patients with gynecological cancer leads to a decrease in the population of viable *Lactobacillus* spp. and *Bifidobacterium* spp. counts [178]. The World Gastroenterology Organization has conducted studies on the prevention of ARE. The Global Guidelines mention both a liquid format (*Lactobacillus casei* DN-114 001) and a powder format (VSL#3) as options for prevention. VSL#3 sachets contain a combination of *Streptococcus thermophilus*, four Lactobacillus species, and three Bifidobacterium species, totaling 450 billion colony-forming units (CFU) [223]. A comprehensive investigation conducted by Delia et al. [167] involved 409 patients who received abdominal radiation after surgery for abdominal and pelvic malignancies. The treatment group was administered VSL#3 during irradiation. The statistically significant findings showed that patients who took VSL#3 had a reduced incidence of diarrhea and fewer episodes of grade 3–4 diarrhea compared to the placebo group. Additionally, the VSL#3-treated patients required lower doses of loperamide. However, some experts believe that based on the available data, VSL#3 is not effective in eliminating ARE. In a multicenter, randomized, double-blind study by Giralt et al. [179], the effects of a liquid format were examined. In the therapy group, patients received 96 ml of a fermented yogurt beverage containing 108 CFU/g *L. casei* DN-11,400 three times daily during the radiation treatment. Based on the Common Toxicity Criteria Grade, the probiotics-treated group did not show improvement in radiation-induced diarrhea. However, a positive effect was observed in terms of stool consistency and reduced frequency of bowel movements among patients in the treated group compared to the placebo group. The results regarding the use of probiotics to prevent ARE are limited and inconclusive. Delia and colleagues [167, 180] found favorable benefits with the combined consumption of different probiotic strains, higher dosages, or a combination of both, in contrast to the findings reported by Giralt and colleagues [179]. Concerning this, Demers and colleagues [177] recently demonstrated that a standard twice-daily dose of Bifilac probiotics (*L. acidophilus* LAC 361 and *B. longum* BB-536; 1.3 billion CFU) could reduce radiation-induced second-, third-, or fourth-grade diarrhea at the end of therapy in individuals with pelvic cancer. To prevent ARE in women undergoing abdominal radiation for gynecologic cancer, Garcia-Peris and colleagues [176] utilized two prebiotics. Their study showed that combining inulin and fructo-oligosaccharides during radiation led to a faster recovery of *Lactobacillus* spp. and *Bifidobacterium* spp. counts two weeks after the completion of radiotherapy, compared to the placebo group [178]. The findings of Garcia-Peris and colleagues [176] were similar to those of Giralt et al. [179] and Demers et al. [177] despite the small sample size. Consequently, Garcia-Peris and colleagues [176] found that the intervention group experienced a reduction in stool consistency (measured on the Bristol scale), but there was no change in the quantity of stools in either group. The administration of a combination of inulin and fructooligosaccharides in patients receiving abdominal radiation is safe and improves their quality of life [176]. However, further studies with larger samples are needed in this area. Additionally, a positive impact was observed in the restoration of intestinal microbiota compared to the placebo group, which may play a crucial role in preventing antibiotic-associated diarrhea [176].

Finally, recent advancements in next-generation probiotics offer promising therapeutic avenues for mitigating such adverse effects. Next-generation probiotics refer to newly identified strains of beneficial microbes, often isolated from healthy human microbiota, with specific and enhanced health benefits. These next-generation probiotics go beyond the traditional *Lactobacillus* and *Bifidobacterium* species, encompassing a broader range of bacteria with unique capabilities to restore and maintain gut health. He et al. [181] study highlighted the pivotal role of *Akkermansia muciniphila* in preserving the integrity of the intestinal barrier and protecting against irradiation-induced intestinal damage. *A. muciniphila*, known for its resilience to oxygen and ability to survive under microaerobic conditions, is more competitive than strict anaerobes in the gut environment. This advantage is crucial, especially in conditions like irradiation, where gut flora diversity and abundance significantly decrease, leading to dysbiosis. These findings confirm that irradiation substantially reduces the diversity and abundance of intestinal flora, with *A. muciniphila* being the most affected. Notably, *A. muciniphila* abundance negatively correlated with diarrhea duration in irradiation-cervical cancer patients, highlighting its potential role in mitigating irradiation-induced GI symptoms. The administration of *A. muciniphila* alleviated intestinal injury and improved the survival rates of irradiated mice, validating its protective role. Mechanistically, *A. muciniphila* degrades mucins in the intestinal mucus layer to produce SCFAs, such as propionate. This process supports the host’s epithelial cells by providing energy sources for synthesizing mucus proteins, thereby maintaining the mucus layer’s integrity. He et al. [181] study showed that *A. muciniphila* enhances mucus production by stimulating goblet cells, which results in a thicker mucus layer, strengthening the intestinal barrier, and reducing irradiation-induced damage. Furthermore, SCFAs like propionate play a critical role in epithelial repair and metabolic regulation. These results indicate that propionate, produced by *A. muciniphila*, mediates its protective effects by promoting the expression of tight junction proteins occludin and Zonula occludens (ZO)-1 through the GRP43 receptor [181]. This mechanism underscores the importance of SCFAs in maintaining the epithelial barrier and preventing pathogenic bacterial invasion. Besides, the discovery of Amuc_1409, a previously uncharacterized protein secreted by *A. muciniphila*, adds a new dimension to our understanding of how this bacterium promotes intestinal health. Kang et al. [182] study revealed the biological functions of Amuc_1409 in enhancing intestinal stem cell (ISC) proliferation and regeneration, particularly in contexts of radiation or chemotherapy-induced intestinal injury. Also, Lapiere et al. [183]. investigated the prophylactic potential of *Faecalibacterium prausnitzii*, a next-generation probiotic, in mitigating acute radiation-induced colonic lesions. The administration of *F. prausnitzii* A2-165 strain before and after irradiation showed significant protective effects against radiation-induced colonic epithelial damage in a preclinical rat model [183]. The probiotic treatment reduced para-cellular hyperpermeability, which is indicative of a strengthened intestinal barrier. Of note, the beneficial effects of *F. prausnitzii* were associated with increased production of IL-18 by colonic crypt epithelial cells [183]. IL-18 is known for enhancing epithelial barrier function and promoting immune responses that protect against gut pathogens. The elevation of IL-18 suggests that *F. prausnitzii* not only aids in physical barrier maintenance but also enhances the immune landscape of the gut, providing a multifaceted defense mechanism against radiation-induced damage. Next-generation probiotics offer a promising therapeutic strategy for managing radiation-induced colitis by restoring microbial diversity, enhancing SCFA production, modulating immune responses, and strengthening the mucosal barrier. As research progresses, these next-generation probiotics could become integral components of supportive care for patients undergoing radiation therapy, improving their quality of life and treatment outcomes.

#### Potential of probiotics against neuronal toxicity

Both human and animal studies have demonstrated that probiotics can improve cognitive deficits and alleviate depression by influencing the gut-brain axis [184]. Furthermore, probiotics are being investigated for their potential to treat neurological conditions such as Alzheimer’s disease and Parkinson’s disease [185, 186]. Radiation exposure compromises gut integrity, allowing bacteria to cross the BBB more easily and spread throughout the body [187]. Additionally, imbalances in gut bacteria can disrupt the gut-brain axis interactions, leading to increased oxidative damage, inflammatory processes, and disruptions in metabolism and immunity.

Studies have shown that ionizing radiation can cause neuroinflammation and alter the structure of the mouse brain, either directly or indirectly, through the gut-brain axis [188, 189]. To communicate with the brain, bacteria utilize various channels, including the vagus nerve, specific neurotransmitters, SCFAs, cytokines, tryptophan compounds, and hormones [190]. The gut microbiota in the host’s digestive tract has a bidirectional communication system known as the gut-brain axis [191]. Moreover, radiation has been linked to increased neuroinflammation, disruption of hippocampal neurogenesis, and damage to neural precursor cells in the dentate gyrus, all contributing to cognitive impairment [192]. A study conducted by Venkidesh and colleagues [187] demonstrated that radiation on whole-body significantly reduced the number of neuronal cells in the brain’s hippocampus region. However, a probiotics formulation (*L. acidophilus*,* L. rhamnosus*,* L. casei*,* Lactobacillus bulgaricus*,* Lactobacillus plantarum*,* B. longum*,* Bifidobacterium breve*,* B. infantis*, and *Streptococcus thermophilus*) showed the ability to decrease the number of pyknotic cells and reduce neuroinflammation by decreasing the number of microglial cells. This finding aligns with earlier research demonstrating how probiotics can mitigate damage and regulate neuroinflammation. Another investigation revealed that pretreatment with a probiotic composition decreased chronic stress-induced abnormal brain plasticity, enhanced neurogenesis, and reduced oxidative stress by enhancing antioxidant enzymes [184, 191, 193]. Probiotics have also been effective in promoting neurogenesis and treating memory impairments [194]. In recent years, probiotic products have successfully demonstrated the neuroprotective capabilities of the probiotic Lab4b in a mouse model of Alzheimer’s disease [195]. Notably, supplementation with *Lactobacillus sp.* has been shown to reduce neuroinflammation in mice [196, 197]. Researchers Rahmati and colleagues found that administering probiotics to mice with cerebral hypo-perfusion improved spatial memory and reduced neuronal damage in the hippocampus [198]. Interestingly, Xueqin and colleagues [199] demonstrated that the probiotic combination ProBiotic-4 alleviates cognitive deficits in geriatric Senescence Accelerated Mouse-Prone 8 (SAMP8) mice by modulating the microbiota-gut-brain axis, suggesting that studying the gut microbiota may be beneficial for managing cognitive dysfunction.

Thus, the existence of the gut-brain axis and the protective qualities of gut flora in the stomach and brain open up new possibilities for innovative treatment approaches to radiation-induced brain damage [147]. It is intriguing to note that individuals with brain tumors have a lower diversity of microbial species in their gut [200]. Probiotics have also shown promise in reducing cancer, particularly glioma, which is the most common malignant tumor of the central nervous system, accounting for 81% of such tumors. Further investigation into the underlying mechanisms of probiotics-induced recovery from neuronal injury and the role of inflammation-related molecules and microbial metabolites could contribute to the development of probiotics for mitigating radiation-induced neurotoxicity.

### Dietary interventions against radiotherapy injury

Recent research has provided evidence that dietary changes can be beneficial in reducing the side effects of radiotherapy [201]. One promising approach is adopting a fiber-rich diet, as it can positively influence the composition and activity of the gut microbiota, leading to improved gut barrier function [202]. Studies have indicated that a high-fiber diet can potentially reduce inflammatory processes and oxidative stress, which are commonly associated with damage caused by radiotherapy [203, 204]. In experiments conducted on mice, those given a high-fiber diet before and after radiation exposure exhibited reduced intestinal damage and decreased inflammation [203].

#### Intestine

Guiqi Baizhu decoction (GQBZD), a Traditional Chinese Medicine (TCM), has been proven effective in reducing colitis symptoms in both mouse and human models [205]. It has also demonstrated significant anti-inflammatory effects in rat studies. Zhang and colleagues [205] utilized a rat model of radiation-induced proctitis, which mimics the condition in humans by locally injuring the rectum through abdominal irradiation. They observed considerable alterations in the diversity and abundance of the gut microbiota in the irradiated rats. Similar changes have been observed in various animal models, including *Bactrocera dorsalis*, *Bank voles*, *Gottingen minipigs*, *Rhesus macaques*, mice, and humans [206–208]. Consistent with previous research by Casero and colleagues [208], the overall abundance of *Firmicutes* decreased, while *Bacteroidetes* increased significantly at the phylum level in the irradiated rats. These changes were reversible with GQBZD treatment. An imbalance between *Bacteroidetes* and *Firmicutes* is a characteristic feature of gut microbiota dysbiosis. Zhang et al. [205] found that irradiated rats had higher relative abundances of *Bacteroidetes* and *Proteobacteria* but lower relative abundances of *Firmicutes* and the *Firmicutes*/*Bacteroidetes* ratio. GQBZD effectively reversed these alterations in *Proteobacteria*, *Firmicutes*, *Bacteroidetes*, and the *Firmicutes*/*Bacteroidetes* ratio.

In a study by Zhang et al. [205], they assessed the phosphorylation of P65. They indicated phosphorylation sites on residue P65 are within the S276 region of the N-terminal active region. When serine 276 is phosphorylated, P65 is activated, leading to increased inflammation downstream in the irradiated (exposure to the abdomen of the rats) group. GQBZD demonstrated the ability to inhibit P65 phosphorylation. The modulatory effect of GQBZD on the gut microbiota may be a key factor in its effectiveness in treating metabolic syndrome and radiation-induced systemic inflammatory conditions, as indicated by their research. Further investigation of the gut bacteria is recommended to validate the effects of GQBZD on the gut microbiota. The NF-κB signaling pathway is likely one of the mechanisms underlying the regulation of the intestinal microecology by GQBZD in the study conducted by Zhang et al. [205]. In conclusion, rats exposed to radiation exhibited changes in the gut microbiota’s metabolites and biological processes, leading to increased intestinal permeability, elevated inflammation, and weakened immune response. GQBZD alleviates radiation-induced inflammation in rats by modulating the gut microbiota composition, suppressing P65 activation, and reducing the expression of inflammatory mediators. While current research focuses on gut microbiota, the question of “who” GQBZD benefits and “how” it functions remains an intriguing issue that warrants further investigation.

Spirulina platensis (SP), a natural microalga known for its rich nutrient content, has been extensively produced and transformed into nutritional supplements[209]. This easily digestible microalga has demonstrated antioxidative, anti-inflammatory, and gut microbiota modulation properties when consumed orally, making it potentially beneficial for preventing and treating various intestinal ailments [210–212]. Furthermore, SP shows promise as a vehicle for drug delivery in intestinal diseases and serves as a versatile micro-carrier for small-molecule medications [213, 214]. Building on these properties, Zhang and colleagues [215] aimed to develop an oral delivery system that could address the unique challenges of maintaining gut microbiota balance and protecting the entire intestine during cancer radiation. As a micro-carrier ranging from 200 to 500 nm, SP exhibited favorable retention within the intestinal villi (300–600 nm) and gradually degraded throughout the GI tract. This allowed SP to distribute drugs across the ileum and small intestine efficiently. Consequently, SP@AMF (SP-loaded mesoporous silica nanoparticles) enhanced the radioprotection of the entire small intestine, particularly the ileum, while extending the radioprotective effects of AMF (amifostine) in vitro. Although enteric-soluble AMF capsules (Cap@AMF) could also protect the medication from gastric destruction, SP@AMF showed a superior protective effect in the distal small intestine, potentially due to the reduced and uneven distribution of medication in the capsules. These findings underscored the suitability of SP as a targeted drug delivery method for the small intestine [215]. The potential explanation for this phenomenon could be attributed to the diminished and erratic medication dispersion within the capsules’ GI tract. These findings showed that the small intestine-targeting medication delivery method SP is more appropriate.

Zhang et al. [215] performed a study on the gut microbiota and made an interesting finding. They discovered that the use of SP@AMF positively impacted maintaining a healthy gut microbiota in mice exposed to radiation. Due to its prebiotic content, including cellulose and polyunsaturated fatty acids, SP has previously demonstrated positive effects on gut flora when consumed orally [216, 217]. The combined benefits of SP in supporting a healthy microbiome may assist in protecting the intestines of cancer patients from radiation-induced damage. The precise function of individual gut microorganisms in radiation-induced intestinal damage has yet to be determined. However, Zhang et al. [215] observed modest alterations in the microbiota composition that were not fully explained. Therefore, future research should employ comprehensive analyses that integrate metagenomics, metabolomics, and transcriptomics to gain a deeper understanding. Long-term safety investigations suggest that SP@AMF could help avoid the toxicity associated with AMF, which is crucial for its clinical application in radiotherapy, as a typical fractionated radiation treatment course lasts at least four weeks. In conclusion, Zhang and colleagues [215] successfully developed SP@AMF, an oral delivery method based on a natural micro-carrier that effectively protects the healthy gut from radiation-induced damage. The inclusion of SP as a natural micro-carrier significantly enhanced the radioprotection provided by AMF throughout the entire gut, thanks to its efficient intestinal biodistribution. Due to its apparent superiority compared to conventional commercial encapsulation techniques, the potential benefits of SP@AMF in promoting a balanced gut microbiota and ensuring long-term safety make it a highly promising candidate for clinical implementation in the context of radiation therapy for abdominal/pelvic malignancies.

#### Brain

Recent research proves that dietary changes can help mitigate radiation-induced brain impairment [218]. Various dietary therapies and microbiota-based techniques have been investigated for their potential to alleviate the side effects of radiation treatment on the brain. In a study conducted by Hu and colleagues [219], it was observed that CNS damage occurred shortly after radiation exposure due to the activation of microglia, altered morphology, activation of injured nerve transcription factors, and production of inflammatory mediators. Following brain radiation exposure, the mice exhibited reduced curiosity, anxiety, and depressive behavior. Pathological changes were observed in the hippocampus’s CA1, CA3, and DG regions. Signaling pathways from the brain to the microbiota, such as the hypothalamic-pituitary-adrenal axis, immune regulation, and vagus nerve stimulation, play a crucial role in this process. The microbiota serves as a protective shield against pathogens in the gut [219]. Alterations in the gut microbiota composition can disrupt intestinal barrier integrity, leading to increased intestinal permeability and the translocation of toxins across the damaged intestinal wall into the bloodstream. The gut microbiota underwent structural changes in a mouse model of radiation-induced brain damage, and the relative abundance significantly decreased. Treatment with quercetin inclusion complex gels (QICG) resulted in an increase in the abundance of *Bacteroidota*. Mice treated with QICG exhibited improved behavior and intestinal barrier function, along with reduced brain and GI damage. QICG did not affect the diversity or abundance of the gut microbiota in antibiotic-treated mice, nor did it alleviate their anxiety levels or pathological alterations. Therefore, the effectiveness of QICG in treating radiation-induced brain damage is largely dependent on gut bacteria. Hu and colleagues [219] successfully synthesized an oral gel formulation of quercetin inclusion complexes, which demonstrated reliability in terms of safety and efficacy through various experiments. By decreasing *Firmicutes* and increasing *Bacteroidota*, QICG modulated the relative richness and diversity of the gut microbiota. By regulating the microbiota-gut-brain axis, QICG reduced GI inflammation and permeability, mitigated irradiation-induced damage to the hippocampus, and improved behavioral cognition, particularly regarding exploration, short-term memory, and anxiety levels. Effective control of the gut microbiota is essential for the success of this therapy. QICG introduces a novel concept and a promising approach for the treatment of radiation-induced brain damage. Future research should further investigate the precise mechanisms of the microbiota-gut-brain axis, including the modulation of the neurological and endocrine systems.

Lycium barbarum (LBE) can enhance specific probiotics, maintain their advantageous presence in the gut over time, and create a favorable bacterial environment for radioprotection. LBE supplementation led to an increase in indole derivatives, metabolites produced by the GI microbiota from dietary tryptophan and known to have immunological significance, as observed in untargeted metabolomics. Previous studies have highlighted the role of indoles in supporting type 3 innate lymphoid cells, which play a crucial role in antimicrobial defense, tissue damage repair, acute phase response, and barrier integrity. Indole metabolites can also enhance IL-1β expression in epithelial cells and increase the expression of the IL-10 receptor on intestinal epithelial cells in an AhR-dependent manner. Additionally, indoles have been shown to protect against medication-induced enteropathy by reducing neutrophil infiltration [220–222]. LBE supplementation also significantly increased the metabolite N-ornithyl-L-taurine. Taurine is involved in various physiological processes such as bile acid conjugation, antioxidant defense, calcium homeostasis, detoxification, neuromodulation, and protection against radiation-induced brain damage in male rats over time [223, 224]. Metabolic pathway analyses revealed enrichment in one-carbon metabolism in the LBE treatment group at days 7 and 14 post-total body irradiation (TBI). These findings indicate that microbiota-based techniques and dietary interventions hold promise in mitigating the adverse effects of radiation therapy on the brain. However, further research is needed to determine these therapies’ optimal dosage, duration, and long-term effects on the microbiota and overall health. It is imperative to prioritize seeking medical counsel prior to implementing any alterations to one’s diet or incorporating supplements, particularly in the context of cancer treatment.

### Microbial metabolites against radiotherapy injury

Although a growing body of evidence has emphasized the significance of gut microbiota-derived metabolites in pathophysiology and immune-mediated homeostasis, the characterization of metabolites and regulatory networks associated with the interplay between the host and microbiota in radiation proctopathy (RP) remains incomplete [286]. Ge and colleagues [225]. Ge and colleagues [226] utilized a mouse model of RP and multi-omics approaches to investigate the dynamics of gut microbiota-derived metabolites and their communication with the host immune system. They aimed to uncover the underlying mechanisms of potential metabolites and their generating bacteria involved in radiation-induced damage. Their results offer new insights into the causative mechanisms underlying the interaction between RP damage, metabolites originating from the gut microbiota, and host immunity. This knowledge may eventually contribute to developing preventive and treatment protocols for RP. The researchers demonstrated that GI inflammation and tissue injury are mediated by 3-hydroxybutyrate (3HB), a metabolite generated by the gut microbiota. In RP mice, administration of 3HB through gavage significantly reduces the expression of G-protein-coupled receptor (GPR) 43, which is a factor in radiation-induced damage [226]. Additionally, their investigation revealed that the gut microbiome pattern in RP mice is characterized by a decrease in core species, particularly *A. muciniphila*, which plays a significant role in determining the level of 3HB. Ge et al. [226] ‘s findings confirmed that the administration of *A. muciniphila* increases the concentration of 3HB in mice and significantly enhances radioprotection. These discoveries advance understanding the relationship between gut flora and the RP disease process, offering potential avenues for mitigating clinical radiation-induced damage through prophylaxis and therapy. The researchers also observed that radiation can alter the metabolite profiles in the feces and urine of RP mice, leading to a significant decrease in the content of 3HB. Previous research has demonstrated that the expression of IL-6 is associated with the extent of radiation-induced damage, making it a useful diagnostic marker for RP [227]. While 3HB has been found to have substantial therapeutic benefits in treating colon cancer and colitis, its role as an immunological effector is still uncertain [228, 229].

Ge et al. [226] confirmed that 3HB directly suppresses radiation-induced IL-6 expression and exhibits radioprotective properties. Earlier studies showed that 3HB therapy reduces the levels of pro-inflammatory cytokines IL-1β, IL-6, and IL-8 in human placental tissue culture, further supporting this finding [230]. Another study demonstrated that 3HB decreases the production of IL-1β and IL-18 by the NOD-like receptor protein 3 (NLRP3) inflammasome in human monocytes [231]. These findings suggest that 3HB acts as an immunological effector and has the potential to serve as a viable facilitator of radioprotection. In addition to 3HB, Ge et al. [226] identified several other metabolites that were decreased in RP mice. Their discovery regarding the role of GPR43 may have therapeutic significance. Previous research has indicated that GPR43 is highly expressed in immunological tissues and cells, highlighting its crucial function in immune responses [232]. Furthermore, Ge et al. [226]’s findings demonstrate the unique relationship between GPR43, gut microbiota, and radiation-induced IL-6 expression in the pathological process of RP. They demonstrated that stimulation of GPR43 inhibits the decreased production of IL-6 by *A. muciniphila* or *A. muciniphila*-mediated 3HB, thereby impeding radioprotection. Overall, these investigations offer a potential clinical alternative for mitigating the damage caused by RP, as current therapeutic techniques for alleviating the adverse consequences of RP are limited and expensive.

Ionizing radiation can generate inflammatory mediators such as IL-1β, TNFα, and NF-κB [233]. SCFAs have been found to exhibit anti-inflammatory properties and can impede cytokine production, which is known to initiate inflammatory responses[233, 234]. Multiple studies have suggested that butyrate can block the NF-κB signaling pathway by protecting the redox mechanism, regulating ROS responsible for NF-κB activation. Additionally, butyrate reduces inflammation by activating Peroxisome proliferator-activated receptor gamma (PPAR-γ), which is widely expressed in colon epithelial cells, and by blocking IFN-γ signaling [235]. Butyrate also inhibits the activation of downstream signaling pathways such as VEGF and Signal transducer and transcription 3 (STAT3) activator by inhibiting HDACs, thereby exhibiting anti-inflammatory effects [236]. Furthermore, SCFAs have been shown to have anti-tumor effects through various signaling pathways, including Wnt/β-catenin, PI3K/Akt/mTOR, MAPK (p38, JNK, and ERK1/2), and EGFR/Kras/Braf [233].

Intestinal fibrosis is a common late side effect of radiation therapy [237]. Kerem and colleagues [238] demonstrated that in rats exposed to radiation, the activity of matrix metalloproteinase-2 was reduced by both the oral administration of soluble fiber and the transrectal administration of SCFAs, leading to improved healing of colon anastomosis. Their findings also indicated that dietary pectin could significantly reduce radiation-induced intestinal fibrosis. By causing protein hyper-acetylation, transcriptional activation, chromatin remodeling, and suppression, as well as cell cycle arrest and cell death, SCFAs can regulate gene expression as HDAC blockers. Chung et al. [239] demonstrated that SCFAs acting as HDAC blockers can decrease the production of radiation-induced TGF-β and tumor TNF-α, enhance skin fibrosis repair, and reduce carcinogenesis and skin fibrosis. As HDAC blockers, SCFAs regulate epithelial-mesenchymal transition (EMT) and intestinal fibrosis, as shown by Wang et al. [240] found that sodium butyrate inhibits cell migration, invasion, and TGF-β-induced EMT in hepatoma cells. These findings indicate that sodium butyrate can effectively mitigate the process of EMT. Furthermore, research by Yang and colleagues [241] also showed that EMT contributes to radiation-induced intestinal fibrosis. This effect is attributed to its ability to regulate intestinal flora and maintain an optimal concentration of SCFAs. Overall, these studies provide valuable insights into the potential therapeutic effects of sodium butyrate and soluble dietary fiber in inhibiting EMT and mitigating intestinal fibrosis caused by radiation. These findings offer promising avenues for the prevention and treatment of radiation-induced complications.

### Antibiotics and radiotherapy injury

According to the research conducted by Zhao et al. [242], pre-treatment with an antibiotic cocktail (Abx) can significantly expedite the restoration of gut microbiota in mice after radiation exposure. Furthermore, Abx pretreatment improved the survival rate of the mice by regulating the LPS/TLR4/MyD88/NF-κB p65/macrophage polarization/TGF-β1/Smad-3 signaling pathway, thereby reducing radiation-induced damage in the GI tract. In their study, the researchers compared the gut microbiota of mice before and after radiation using 16 S rRNA sequencing technology. Their findings supported the sequencing of gut microbiota in patients with radiation-induced intestinal diseases, demonstrating that abdominal radiation disrupted the gut microbiota and reduced intestinal microbiota diversity in mice [243]. Based on the research conducted by Zhao et al. [242], it was observed that pretreatment with antibiotics successfully eradicated a significant portion of the intestinal microbiota found in the feces of mice, thereby replicating the GI conditions observed in germ-free mice. The study indicated that the control group had fewer unique OTUs compared to the antibiotic pretreatment groups. Additionally, the abundance of *Verrucomicrobia* was significantly higher in the Abx pretreatment group than in the control group. The abundance of *Verrucomicrobia* was also increased, and the restorative effect of metronidazole (MDE) pretreatment on intestinal microbiota following radiation was superior to that of the control group [242]. Abx pretreatment enhanced the ability of the gut microbiota to reestablish itself after radiation exposure, leading to higher diversity compared to the control group. Although MDE pretreatment worsened the mice’s gut microbiota problem, it facilitated the recovery of the intestinal microbiota following radiation-induced damage and increased the diversity of the rebuilt microbiota.

According to the findings of Zhao et al. [242], pretreatment with Abx significantly increased the long-term survival rate of mice following abdominal radiation, whereas the control group and MDE pretreatment groups had higher mortality rates. Furthermore, the Abx pretreatment group mice showed greater proliferative capacity and apoptotic capability in their intestinal epithelial cells at 1 month and 3 months after radiation, as evidenced by the protein expression of PCNA and cleaved caspase3. The increased apoptosis and cell growth at these time points suggested that Abx pretreatment facilitated the healing and regeneration of intestinal epithelial cells. In this study, the mice pretreated with Abx had significantly lower levels of LPS in their ileum compared to the control group. Previous research has shown that pretreatment with a TLR4 antagonist in mice reduces radiation-induced cell death and damage [244]. In the study conducted by Zhao et al. [242], the mice subjected to Abx pretreatment exhibited a significant decrease in the abundance of TLR4 in their ileum. Additionally, the expression of MyD88 and phosphorylated NF-κB p65 proteins was decreased in the Abx pretreatment group. Recent studies have indicated that controlling radiation-induced cardiac senescence heavily depends on the macrophage migration inhibitory factor [245]. In a study conducted by Zhao et al. [242], it was observed that the protein expression levels of CD163, a marker for M2 macrophages, and inducible nitric oxide synthase (iNOS), a marker for M1 macrophages, were significantly reduced in the ileum of the group that received Abx pretreatment, as compared to the control group. It is believed that an increase in the number of macrophages in the lamina propria leads to intestinal fibrosis, and cytokines secreted by macrophages, including TGF-β1, can promote the accumulation and fibrosis of fibroblasts and extracellular matrices [246]. Even 26 weeks after radiation treatment, TGF-β1 remained at high levels in smooth muscle, endothelial cells, and lamina propria fibroblasts [247]. According to the findings of Zhao et al. [242], the administration of Abx prior to treatment decreased the expression levels of proteins, including TGF-β1, phosphorylated Smad-3, and α-SMA. This observation suggests that Abx may have a potential therapeutic effect in mitigating intestinal wall fibrosis in mice exposed to radiation, possibly by inhibiting the TGF-β1/Smad-3 signaling pathway. In conclusion, our findings demonstrate that abdominal radiation affects the intestinal microecology in mice, reducing microbial diversity and increasing the relative abundance of harmful bacteria such as Proteobacteria. Pretreatment with Abx and MDE helps restore the gut flora in radiated mice. Furthermore, Abx pretreatment improves the survival of mice with post-radiation intestinal injury by modulating the LPS/TLR4/MyD88/NF-κB p65/macrophage polarization/TGF-β1/Smad-3 signaling pathways. These findings suggest a potential therapeutic approach for animals at risk of intestinal damage from abdominal radiation.

### Fecal microbiota transplantation to reduce radiotherapy injury

Patients with compromised or imbalanced gut microbiota may undergo FMT, a procedure where feces from a healthy donor are transplanted into their GI tract [248]. FMT has proven beneficial in treating recurrent *Clostridioides difficile* infection and IBD [249]. While there is limited specific information on using FMT to mitigate radiation-induced injury, certain studies suggest that it may improve the gut microbiota and reduce intestinal inflammation, potentially reducing the extent of toxicity caused by radiotherapy in the GI tract.

Enhancement of the gut microbiota through FMT may improve GI function and preserve epithelial integrity in radiation-induced enteritis while also reducing intestinal permeability [250]. Ding and colleagues [19] found that all patients who received FMT after radiation treatment exhibited a more diverse gut flora. After eight weeks of FMT, radiation-induced intestinal edema was noticeably reduced, and the abundance of beneficial bacteria such as *Alistipes*, *Phascolarctobacterium*, *Streptococcus*, and *Bacteroides* increased, while Faecalibacterium was less prevalent. FMT using gut microorganisms from healthy donors may help mitigate radiation damage [250]. In irradiated mice that underwent FMT, higher levels of microbially derived indole 3-propionic acid (IPA) were observed in their feces, which reduced chronic inflammation, decreased the risk of hematopoietic organ damage and myelosuppression, and improved GI function and epithelial integrity [251]. Similarly, Li et al. [144] reported that FMT increased the concentration of SCFAs in feces, with valeric acid generated by the microbiota having the most significant radioprotective effect by increasing the expression of keratin 1 (KRT1). Supplementation with valeric acid helped irradiated mice live longer, preserve their hematopoietic organs, and reduce GI damage [144].

In a study, Wang et al. [252]. found that FMT is an effective treatment for chronic radiation enteritis, offering significant symptom relief by altering the gut microbiota composition. The transition from a pathogenic to a beneficial bacterial profile correlates with the alleviation of clinical symptoms, emphasizing the therapeutic potential of FMT. The patient’s clinical symptoms were significantly alleviated following two courses of FMT, indicating the procedure’s short- and long-term efficacy [252]. The electronic colonoscopy confirmed the diagnosis of chronic radiation enteritis, and subsequent 16 S rRNA microbiological analysis provided insights into the changes in the patient’s gut microbiota. Before FMT, the patient’s intestinal flora was dominated by pathogenic bacteria, such as *Escherichia fergusonii* and *Romboutsia timonensis*, which are associated with GI distress and inflammation [252]. In conclusion, further research is necessary to fully understand the role of FMT in mitigating radiation-induced damage in individuals.

## Microbiota as a predictive biomarker of radiotherapy adverse effects

Several studies have investigated the potential use of microbiota as a biomarker for radiation side effects. Wang and colleagues [253] conducted research to evaluate inflammatory markers, fecal microbial ecology, and symptoms of fatigue and diarrhea in cancer patients undergoing a five-week pelvic radiation course. Their findings suggested that individuals who are more susceptible to developing diarrhea after pelvic irradiation may exhibit pre-existing alterations in gut microbial ecology [253]. Notably, changes in the gut’s microbial ecology prior to pelvic radiation were particularly significant. Patients who experienced diarrhea showed significantly reduced microbial alpha diversity and a higher *Firmicutes*/*Bacteroides* ratio compared to those who did not develop diarrhea. Wang et al. [253] also reported significant differences in the taxonomic analysis of pre-radiotherapy patient groups, with distinct relative abundances of *Veillonella*, *Prevotella*, *Parabacteroides*, *Oscillibacter*, *Faecalibacterium*, *Dialister*, *Bacteroides*, and *Clostridia* clusters XI and XVIII. Similar arguments can be made for the *Faecalibacterium* genus, which contains the protective commensal *F. prausnitzii* [254]. Similar arguments can be made for the *Faecalibacterium* genus, which includes the protective commensal *F. prausnitzii* [255]. Conversely, *Clostridium* cluster XI, which consists of the well-known diarrheal pathogen *C. difficile*, exhibited a substantial increase after radiation in patients who developed diarrhea but showed the opposite pattern in patients who did not experience diarrhea. In conclusion, Wang et al. [253] found a strong association between gut microbial dysbiosis, fatigue, and diarrhea after pelvic irradiation, supporting the need for larger-scale efforts to identify more accurate predictors and preventive measures for intestinal radiotoxicity.

Radiation-induced pulmonary damage has become a common and dose-limiting complication in the treatment of thoracic cancers such as breast cancer and lung cancer [256]. This condition is clinically diagnosed, permanent, incurable, and can worsen patients’ prognoses, potentially leading to death [257]. Consequently, numerous studies have focused on identifying potential biomarkers that can diagnose and predict the prognosis of radiation-induced lung damage. Guo and colleagues [256] investigated the composition and anticipated function of the lung microbiome in rats with radiation-induced pulmonary damage, providing valuable insights. They proposed that *Escherichia-Shigella*, *Bifidobacterium*, *Lactobacillus*, and *Parabacteroides* may serve as bacterial biomarkers and potential targets for the therapy or detection of radiation-induced lung damage. However, further research is needed to verify whether these specific microbial communities indeed influence the onset and progression of radiation-induced lung damage.

A study conducted by Zhu and colleagues [110] examined the progression and grading of oral mucositis in patients with NPC undergoing radiation therapy. The patients’ oral mucosa was examined and sampled eight times throughout the trial period. The study found that healthy controls had more diverse and comparable bacterial populations compared to NPC patients. The impact of radio-chemotherapy on the oral microbiota is well-documented, resulting in compromised innate immune responses and observable alterations in oral flora [258]. To more accurately represent the dynamic shifts in mucosal microbiota, the retropharyngeal mucosa of patients was longitudinally sampled in Zhu et al. [110] study as mucosal swab collection provides a more precise representation than mouthwash, dental plaque, and saliva sampling. Surprisingly, the study revealed that individuals in the severe mucositis category had higher levels of *Streptococcus* even before the appearance of erythema. The *Streptococcus* genus consists of over 50 species, many of which are significant human pathogens and also make up a substantial portion of oral commensals [259]. *Streptococcus mitis* levels increased dramatically in both breast cancer and NPC patients after radiation treatment [260, 261]. When combined with other oral Streptococci, S. mitis acts as a mucin-degrading bacterium, which plays a crucial role in mucus breakdown within the oral cavity [262]. As the degradation progresses, the protective function of the epithelium becomes impaired, potentially allowing pathogen translocation into the lamina propria and attracting inflammatory cells [263]. Therefore, it appears that an elevated abundance of *Streptococci* may predispose patients to early-stage oral mucus malfunction, potentially disrupting the ecological balance of the oral microenvironment more severely in the severe mucositis subgroup before erythema develops. From the onset of visible erythema to the development of severe mucositis (RTOG 3), the study by Zhu et al. [110] found that patients in the moderate mucositis subgroup had a more diverse bacterial community and lower levels of *Actinobacillus* compared to those in the severe subgroup. A diverse oral bacterial community is believed to play a significant role in protecting the oral ecology against the overgrowth of native pathobionts, including *Actinobacillus* spp. observed in this study. These species are known to function as both commensals of the oropharynx and opportunistic pathogens frequently involved in the pathogenesis of meningitis, sinusitis, pleural empyema, and bronchopneumonia [264]. *Actinobacillus* spp. can significantly influence oropharyngeal microbial balance and may be a contributing factor that predisposes patients to severe mucositis due to its increased abundance in the severe mucositis group and its commensal/pathogen lifestyle. The findings concluded that the development and progression of radiation-induced mucositis in NPC patients were associated with changes in the oral microbial community, suggesting that microbiota-based strategies could be utilized to predict and prevent severe mucositis during radiotherapy.

## Conclusion

In conclusion, the emerging understanding of the role of the microbiome in cancer opens up intriguing possibilities for enhancing tumor control and mitigating side effects in radiotherapy. However, further research is crucial to unravel the intricate processes involved and establish the causal effects of the gut microbiota, metabolome, and immunology. Exploring alternative dietary fibers or combinations is also necessary to improve the therapeutic ratio of radiation. It is well-documented that radiation exposure can disrupt the immune system, upset the balance of intestinal microbiota, and damage the intestinal and vascular endothelium, leading to radiation enteritis. The gut flora plays a critical role in the etiology and treatment of radiation-induced damage. Of note, radiation exposure has been found to enhance the proliferation of detrimental gut microorganisms, thereby exacerbating the risk of developing intestinal diseases. On the other hand, beneficial bacteria that naturally inhabit the gut can directly impact intestinal epithelial cells or modulate the gut’s immune system, providing radioprotective effects. Given the dual-edged nature of controlling the gut microbiota, it holds great promise for preventing and treating radiation enteritis. This review has highlighted the pivotal role and mechanisms of the gut microbiota and the complex interplay between the microbiota and radiation efficacy and injury. Various microbiome-based therapies, such as probiotics, FMT, antibiotics, microbial metabolites, and natural compounds, have been discussed as potential strategies for managing radiation-induced harm. While these therapies serve as a roadmap for future management of radiation damage, it is important to note that current research indicates radiation adversely affects the gut microbiota, reducing the population of probiotic bacteria and their metabolite expression, thereby exacerbating radiation-induced stomach damage. The continual advancement of sequencing, metabolomics, transcriptomics, shotgun metagenomics, and other cutting-edge techniques is instrumental in unraveling the complexities of the human microbiome. Ultimately, the success of future treatments for radiation-induced damage will rely on integrating microbiota-based therapeutic approaches.

### Electronic supplementary material

Below is the link to the electronic supplementary material.

Supplementary Material 1

Supplementary Material 2

## Data Availability

Not applicable.
