# Supplementary material for: Microbiome in radiotherapy: an emerging approach to enhance treatment efficacy and reduce tissue injury
Source: Mol Med. 2024 Jul 19;30:105. doi: 10.1186/s10020-024-00873-0 (PMC11264922; doi:10.1186/s10020-024-00873-0)
Supplement: Supplementary file 1 — Supplementary Material 1 [file 10020_2024_873_MOESM1_ESM.docx]

MOME-D-23-01334
Microbiome in Radiotherapy: An Emerging Approach to Enhance Treatment Efficacy and Reduce Tissue Injury

Dear Dr. Ruggieri,

Thank you for your valuable feedback and for providing the reviewers' comments on our manuscript, "Microbiome in Radiotherapy: An Emerging Approach to Enhance Treatment Efficacy and Reduce Tissue Injury" (MOME-D-23-01334). We have carefully considered all the suggestions and have made the necessary revisions to address the concerns raised. Below is our detailed response to each reviewer's comments:

Reviewer reports:

Reviewer #1: The topic covered in the review is very interesting and should be discussed.
The authors have made a great effort to select the literature and address the various themes.
However, the review needs to be extensively revised. In fact, some sections are discussed in a specific manner (for example how radiation damage occurs) which instead are concepts that should be considered acquired.
Furthermore, since the focus is on the role of the microbiome in radiotherapy, the data reported should be more specific, the type of tumor, the type of radiation should be reported and the resulting alteration of the microbiota should be specified. In the absence of these specifications, the topics are treated in a general way.

We have revised sections discussing radiation damage mechanisms to ensure these concepts are treated as assumed knowledge.

We have provided more specific data on tumor types, radiation types, and the resulting alterations in the microbiota.

for example lane 148, 156, 245-248 which bacteria?

lane 195, 200-211 which tumor?
lane 315-17 explain how the two strategies involve bacteria
lane 337-349 is radiotherapy also used or just chemotherapy? indicate tumor and x-ray doses)

All the modifications were done and added.

Some concepts are repeated and need to be summarized (section 4 repeats for each organ the concept that dysbiosis is associated with damage to the reference organ. Indicate this in the general paragraph and do not mention it in the subsequent specific sections)
Section 4:
lane 405 indicate the type of tumor
lane 409-10 it is unclear whether it refers to cancer or other skin pathologies
lane 439-441 the study is cited but the result is not specified.
lane 448-449 and 450-453 explain how microbiome diversity is altered.
lane 473 which group develops tooth decay?
lane 519-525 summarize
lane 531specify what is modulated
reference 135 does not appear to be related to treatment with x-ray, in this case eliminate
lane 706 specify the site of the radiation that induces dice to the hippocampus

The modifications were done and added.

Section 5
lane 776 specify which combinations of probiotics have a preventive effect
lane 813, 834, 891, 951 specify probiotic mixture
lane 950, 975, 1013 specify radiation location
paragraph from 1095-1126 why is it included in the brain section?
lane 1141 is it possible that taurine increases epithelial barrier functions by stimulating the production of IL8? maybe the opposite?
lane 1206-7 indicate by what the activity of MMP-2 is inhibited
in the section about FMT the paragraph from 1305-1320 should be deleted or summarized because it is not related to radiation.
Lane 1353 which bacterial genera and species can function as biomarkers?

The modifications were done and added.

Minor points
Lane 272 check the reference numbering. (ref 116 appears after n 58)
lane 352-357 paragraph that is too general I suggest deleting it
lane 527 typo essor 0=T0?
lane 400-403 and 412-414 references are missing

Always specify acronyms the first time they appear:
Lane 570 PC?
AZD6244?
lane 437 HNC?
lane 711 DG and CA2
lane 739 NMDA
Lane 805 insert acronyms for the groups described
lane 837 VLS
lane 1243 MDE
lane 1321 I suggest predictive instead of predation
lane 1338-1339 the sentence is repeated
lane 1358 NPC

The modifications were done and added.

Reviewer #2: Dear Sirs,
This is a very interesting, comprehensive, and well-written review manuscript that sheds light on the human microbiome and its impact on enhancing radiation effectiveness while minimizing damage. I recommend the publication of this manuscript after considering some recommendations and observations to increase the quality of the paper. In particular:

1. The role of next generation probiotics should be further discussed in this manuscript, especially in the case of colitis due to radiation therapy. The authors may comment on findings by:
- He KY, Lei XY, Wu DH, Zhang L, Li JQ, Li QT, Yin WT, Zhao ZL, Liu H, Xiang XY, Zhu LJ, Cui CY, Wang KK, Wang JH, Lv L, Sun QH, Liu GL, Xu ZX, Jian YP. Akkermansia muciniphila protects the intestine from irradiation-induced injury by secretion of propionic acid. Gut Microbes. 2023 Dec;15(2):2293312. Doi: 10.1080/19490976.2023.2293312 and
- Kang EJ, Kim JH, Kim YE, Lee H, Jung KB, Chang DH, Lee Y, Park S, Lee EY, Lee EJ, Kang HB, Rhyoo MY, Seo S, Park S, Huh Y, Go J, Choi JH, Choi YK, Lee IB, Choi DH, Seo YJ, Noh JR, Kim KS, Hwang JH, Jeong JS, Kwon HJ, Yoo HM, Son MY, Kim YG, Lee DH, Kim TY, Kwon HJ, Kim MH, Kim BC, Kim YH, Kang D, Lee CH. The secreted protein Amuc_1409 from Akkermansia muciniphila improves gut health through intestinal stem cell regulation. Nat Commun. 2024 Apr 6;15(1):2983. doi: 10.1038/s41467-024-47275-8.

We have discussed the role of next-generation probiotics, especially regarding colitis due to radiation therapy. Specifically, we commented on findings from He et al. (2023) and Kang et al. (2024).

2.In addition, authors may comment on the findings of Lapiere A, Geiger M, Robert V, Demarquay C, Auger S, Chadi S, Benadjaoud M, Fernandes G, Milliat F, Langella P, Benderitter M, Chatel JM, Sémont A. Prophylactic Faecalibacterium prausnitzii treatment prevents the acute breakdown of colonic epithelial barrier in a preclinical model of pelvic radiation disease. Gut Microbes. 2020 Nov 9;12(1):1-15. doi: 10.1080/19490976.2020.1812867 regarding administration of Faecalibacterium prausnitzii and colitis.

We added comments on the findings of Lapiere et al. (2020) concerning the administration of Faecalibacterium prausnitzii and colitis prevention.
3.Besides, regarding FMT the authors perhaps would like to refer to a very recent reference: Wang L, Li Y, Zhang YJ, Peng LH. Intestinal microecological transplantation for a patient with chronic radiation enteritis: A case report. World J Gastroenterol. 2024 May 21;30(19):2603-2611. doi: 10.3748/wjg.v30.i19.2603.

We added the recent case report by Wang et al. (2024) on intestinal microecological transplantation for chronic radiation enteritis.
