# Supplementary material for: Microbiome in radiotherapy: an emerging approach to enhance treatment efficacy and reduce tissue injury
Source: Mol Med. 2024 Jul 19;30:105. doi: 10.1186/s10020-024-00873-0 (PMC11264922; doi:10.1186/s10020-024-00873-0)
Supplement: Supplementary file 2 — Supplementary Material 2 [file 10020_2024_873_MOESM2_ESM.docx]

*Dr. Betty Diamond*

Editor-in-Chief of ‘*Molecular Medicine’* journal

The Feinstein Institutes for Medical Research, United States of America

Dear Dr. Diamond,

It would be a pleasure to submit the revised manuscript entitled **“Microbiome in Radiotherapy: An Emerging Approach to Enhance Treatment Efficacy and Reduce Tissue Injury”** (**MOME-D-23-01334)** in your journal. Our revised manuscript contains the modifications, as suggested by the reviewers, which are shown in the yellow highlighted. Besides, the response to the comments point-by-point is provided, and we hope these responses and modifications are acceptable to you and the reviewers.

Sincerely,

***Corresponding author**: Dr. Lina Lu, School of Chemical Engineering, Northwest Minzu University, No.1, Northwest New Village, Lanzhou 730030, Gansu, China Tel: 0931-4512930. Email: [lulina1113@163.com](mailto:lulina1113@163.com)
